# Harnessing Nature’s Chemistry: Deciphering Olive Oil Phenolics for the Control of Invasive Breast Carcinoma

**DOI:** 10.3390/molecules30153157

**Published:** 2025-07-28

**Authors:** Nehal A. Ahmed, Abu Bakar Siddique, Afsana Tajmim, Judy Ann King, Khalid A. El Sayed

**Affiliations:** 1 Department of Basic Pharmaceutical and Toxicological Sciences, College of Pharmacy, University of Louisiana at Monroe, 1800 Bienville Drive, Monroe, LA 71201, USA; atefkhaledahmedabdn@warhawks.ulm.edu (N.A.A.); siddique.ulm@gmail.com (A.B.S.); afsana.ulm@gmail.com (A.T.); 2 Foundational and Clinical Sciences Department, Thomas F. Frist, Jr. College of Medicine, Belmont University, 1900 Belmont Boulevard, Nashville, TN 37212, USA; judy.king@belmont.edu

**Keywords:** breast cancer, EVOO, (−)-ligstroside aglycone, metastasis, (−)-oleocanthal, progression

## Abstract

Breast cancer (BC) is the most common malignancy and the second-leading cause of cancer-related mortalities in women. Epidemiological studies suggested the reduced BC incidence in Mediterranean populations due to the daily consumption of diets rich in extra-virgin olive oil (EVOO). EVOO secoiridoid phenolics are widely known for their positive outcomes on multiple cancers, including BC. The current study investigates the suppressive effects of individual and combined EVOO phenolics for BC progression and motility. Screening of a small library of EVOO phenolics at a single dose of 10 µM against the viability of the BC cell lines ZR-75-1 (luminal A) and MDA-MB-231 (triple negative BC, TNBC) identified oleocanthal (OC) and ligstroside aglycone (LA) as the most active hits. Screening of EVOO phenolics for BC cells migration inhibition identified OC, LA, and the EVOO lignans acetoxypinoresinol and pinoresinol as the most active hits. Combination studies of different olive phenolics showed that OC combined with LA had the best synergistic inhibitory effects against the TNBC MDA-MB-231 cells migration. A combination of 5 µM of each of OC and LA potently suppressed the migration and invasion of the MDA-MB-231 cells versus LA and OC individual therapies and vehicle control (VC). Animal studies using the ZR-75-1 BC cells orthotopic xenografting model in female nude mice showed significant tumor progression suppression by the combined OC-LA, 5 mg/kg each, ip, 3X/week treatments compared to individual LA and OC treatments and VC. The BC suppressive effects of the OC-LA combination were associated with the modulation of SMYD2–EZH2–STAT3 signaling pathway. A metastasis–clonogenicity animal study model using female nude mice subjected to tail vein injection of MDA-MB-231-Luc TNBC cells also revealed the effective synergy of the combined OC-LA, 5 mg/kg each, compared to their individual therapies and VC. Thus, EVOO cultivars rich in OC with optimal LA content can be useful nutraceuticals for invasive hormone-dependent BC and TNBC progression and metastasis.

## 1. Introduction

Breast cancer (BC) is the most commonly diagnosed malignancy among females and the second leading cause of cancer-related mortalities in women worldwide. According to statistics, 316,950 estimated new BC cases and 42,170 deaths are expected in the United States this year [1]. A group of manageable and non-manageable factors contributes to the increased risk of developing BC. Manageable risk factors are changeable or avoidable and include obesity, sedentary lifestyle, and exposure to exogenous hormones, while hereditary predisposition and aging are among the non-manageable factors that are non-avoidable [2]. BC is a heterogeneous disease that is classified to four main subtypes: luminal A (ER^+^ and/or PR^+^/HER2^−^/low Ki-67), luminal B (ER^+^ and/or PR^+^/HER2^+^/high Ki-67), non-luminal HER2^+^ (ER^−^ and PR^−^/HER2 overexpressed), and triple negative BC (TNBC, ER^−^, PR^−^, HER2^−^) [3]. Current therapeutic strategies include surgical intervention, radiation, chemotherapies (taxanes, anthracyclines, and platinum-based), hormonal therapy (selective estrogen receptor modulators, SERMs), aromatase inhibitors, targeted therapies (tyrosine kinase inhibitors), in addition to immunotherapies (immune checkpoint inhibitors and monoclonal antibodies) [4,5]. However, these therapeutic options induce severe off-target effects, fail to prevent recurrence, and develop rapid resistance via altered signaling pathways like upregulating the PI3K (phosphatidylinositol 3-kinase), mTOR (mammalian target of rapamycin), and Ras-ERK (extracellular signal-regulated kinase) signaling pathways [6]. Thus, there is a dire need to discover new effective BC therapeutic alternatives.

The SMYD2, SET, and MYND domain-containing protein 2, a lysine methyltransferase, is aberrantly dysregulated in different cancer types, inducing several mitogenic signaling pathways by methylating lysine moieties, activating multiple oncogenic and effector proteins [7,8]. SMYD2 methylates several histone H3K4 and H3K36 and non-histone mitogenic proteins in BC, together with p53, Rb, HSP90, PTEN, and estrogen receptor-α (ERα) [9]. SMYD2 contributes to the progression of TNBC by methylating and activating non-histone protein targets, specifically STAT3 and NF-κB, while also methylating histone substrate proteins, regulating the transcription of numerous BC-associated genes [10,11]. Moreover, the enhancer of zeste 2 polycomb repressive complex 2 subunit (EZH2) is a downstream SMYD2 protein substrate and assumed among the top lysine methyltransferases significantly upregulated in metastatic BC cells [11,12]. SMYD2-mediated EZH2 di-methylation at lysine 307 (K307) activates and elevates its stability, which can be demethylated by the histone H3K4 lysine-specific demethylase 1 (LSD1) in BC [11,12,13]. EZH2-K307 dimethylation promotes the proliferation and invasion of BC cells through facilitating the recruitment of EZH2 to chromatin remodeling and the subsequent transcriptional repression of EZH2 target genes [12]. EZH2 non-canonically methylates STAT3, promoting its nuclear localization [13]. This leads to an overexpression of genes that drive BC proliferation and migration, advancing the progression [13].

The Mediterranean diet (MD) is one of the most widely recognized and extensively studied dietary patterns globally [14,15,16]. MD reflects the traditional high consumption of non-starchy vegetables, seeds, nuts, minimally processed whole grains, legumes, and olive oil, and moderate consumption of dairy products and alcohol, as well as low meat intake [14,15]. Epidemiological studies established the positive impact of sticking to the MD on overall human health, including cardiovascular health, obesity, and a lower incidence of various types of cancer, diabetes, and dyslipidemia [16,17]. Prospective studies have shown that MD can lower overall mortality and has been linked to a slower progression of age-related cognitive decline and a reduced risk of certain malignancies [17,18]. Extra-virgin olive oil (EVOO) is one of the hallmark ingredients of the MD [19,20]. The health benefits of EVOO are attributed to its high content of monounsaturated fatty acids, which can constitute up to 80% of its total fats [20,21]. However, studies indicated that EVOO’s minor phenolic compounds are key players in various health benefits attributed to EVOO consumption [20,21]. EVOO phenolics are grouped into five categories: phenolic acids, alcohols, secoiridoids, lignans, and flavones [21].

*S*-(−)-Oleocanthal (OC) was initially isolated by the Montedoro group from EVOO and was later identified by the Beauchamp group as the potent nonsteroidal anti-inflammatory EVOO ingredient with comparable ibuprofen-like cyclooxygenases (COXs) inhibitory activity [22,23]. OC demonstrated antioxidant and neuroprotective properties, showing positive outcomes in cellular and animal models of Alzheimer’s disease by reducing Aβ-amyloid fibrils and accumulation of neurofibrillary tangles in the brain, and suppressed complement component 3a receptor 1 activity [24]. Furthermore, OC exhibited anti-inflammatory effects by reducing IL-6 expression and inhibiting the release of 5-lipoxygenase [24]. OC showed potential anticancer effects in various cellular and animal models [25]. OC suppressed hepatocellular carcinoma growth and metastasis by inactivating STAT3 both in vitro and in vivo [26]. OC had also been reported to inhibit mesenchymal-epithelial transition factor (c-MET) receptor tyrosine kinase and its downstream signaling pathways, reducing the activation of the mechanistic target of rapamycin (mTOR) levels, and suppressing COX-2 expression in different cancer types [27]. Previously, this study team validated OC as an effective modulator for the ER expression and function in hormone-dependent BC, in both in vitro and in vivo models [28]. OC showed a similar ER binding mode and revealed synergistic effects when combined with the selective ER modulator tamoxifen [28]. The same team also showed that OC significantly synergized with the dual EGFR-HER2 antagonist lapatinib using both in vitro and in vivo model systems of hormone-dependent luminal B BC [29]. A significant TNBC progression suppression was recorded in a nude mouse orthotopic xenograft model upon oral administration of OC nano-emulsion [30]. OC nano-emulsion was selectively in vitro cytotoxic to BC cells but showed modest effects on the viability of the non-tumorigenic mammary epithelial cells MCF-10A [30]. OC effectively suppressed the luminal B BC (BT-474 cells) and TNBC (MDA-MB-231 cells) locoregional recurrence after primary tumor surgical excision in nude mouse xenograft models [31]. A recent study explored the synergy of EVOO phenolics double combinations and crude occurrence in extracted EVOO samples for potential suppression of the proliferation of diverse cancers [32]. Among the discovered most active combinations was the improved anti-luminal A BC activity of an EVOO sample rich in LA and oleuropein aglycone (OA), exceeding the OC and hydroxyoleocanthal (oleacein, HOC) level. This LA-OA-rich EVOO sample inhibited the luminal BC cell line MCF-7 cells’ viability by 50% at 14.8–22.9 µg/mL LA+OA [32]. This activity level was >25-fold higher than the previously reported EC_50_ values (~500 µg/mL) for multiple EVOO total phenolic extracts rich in LA and OA against the MCF-7 BC cell line [32].

Based on the epidemiological studies, promising anticancer outcomes correlating the regular consumption of EVOO with improved human health, and the documented positive anti-BC results of OC, the present study aims at comprehensive in vitro screening of a small olive phenolics library for luminal A BC growth and TNBC migration suppression, followed by selecting the most in vitro active hits for subsequent TNBC anti-metastatic and luminal A progression suppression in vivo activities individually and in combinations. Combination studies can guide future selection of the best EVOO varieties for potential translational nutraceutical use by BC patients and survivors to control their disease and prevent its relapse.

## 2. Results

### 2.1. Analysis of SMYD2, STAT3, and EZH2 mRNA Expression in BC Patients

**Figure 1:**
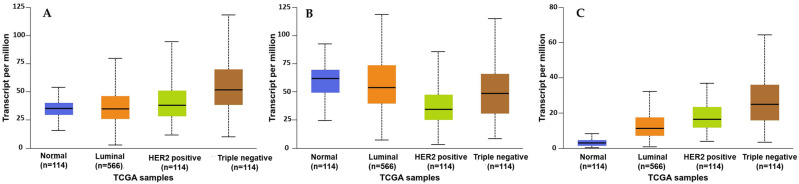
Comparison of the SMYD2 (**A**), STAT3 (**B**), and EZH2 (**C**) normalized mRNA expression levels in normal breast tissues versus the BC subclasses luminal, HER2^+^, and TNBC.

The Human Cancer Genome Atlas (TCGA) database was analyzed using the UALCAN GEPIA to investigate the differential expression patterns of SMYD2, STAT3, and EZH2 across various BC subtypes (Figure 1) [33,34,35,36,37]. These genes were found to be significantly overexpressed in multiple cancer types, including liver, uterine, lung, and prostate cancers (Supplementary Figure S1A–C). Further exploration within one cohort revealed that SMYD2 was significantly overexpressed (*p* = 1.11 × 10^−16^) in primary BC (*n* = 1097) compared to normal tissue samples (*n* = 114). STAT3 was more significant (*p* = 1.05 × 10^−1^) in primary BC (*n* = 1097) versus normal tissue samples (*n* = 114). Similarly, EZH2 expression was more significant (*p* = 1.11 × 10^−16^) in primary BC (*n* = 1097) versus normal tissue samples (*n* = 114) (Supplementary Figure S1D–F).

Analysis of the expression of SMYD2 across BC subclasses showed significant overexpression in luminal (*n* = 566), HER2^+^ (*n* = 37), and TNBC (*n* = 116) compared to normal breast tissues (*n* = 114). A similar expression pattern was also observed for STAT3 and EZH2 expression among BC subclasses (Figure 1A–C). Earlier findings were consistent with another cohort study using GEPIA 2, which further validated the clinical dysregulation of SMYD2, STAT3, and EZH2 in multiple organ malignancies (Supplementary Figure S2A–C). Moreover, another cohort revealed the highest SMYD2 expression in BC tissues (*n* = 1085) versus normal tissue samples (*n* = 112) (Supplementary Figure S2D). A comparable pattern of significance was also observed for STAT3 and EZH2 in BC (Supplementary Figure S2E,F) [33,34,35,36,37].

### 2.2. Suppressive Effects of Olive Phenolics on Proliferation and Migration of Invasive Breast Carcinoma Cells

**Figure 2:**
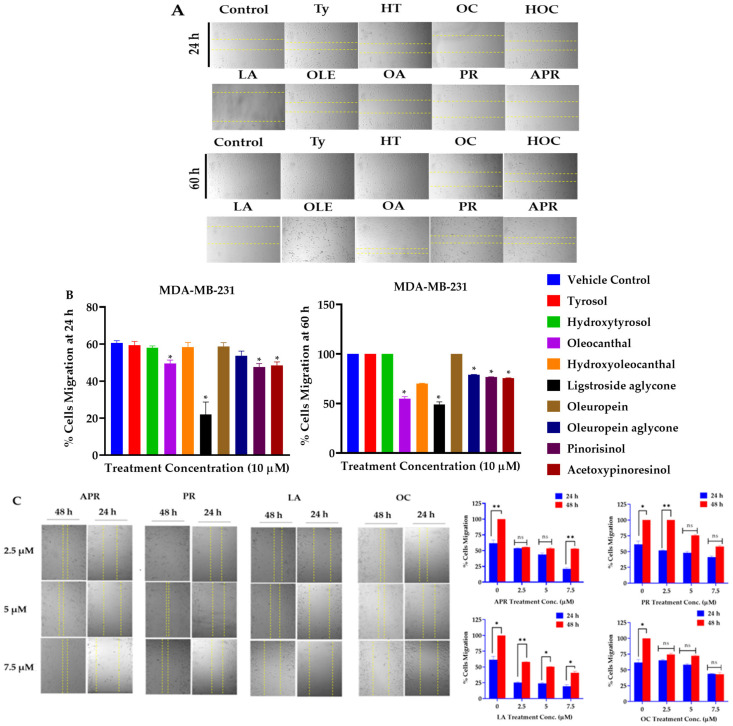

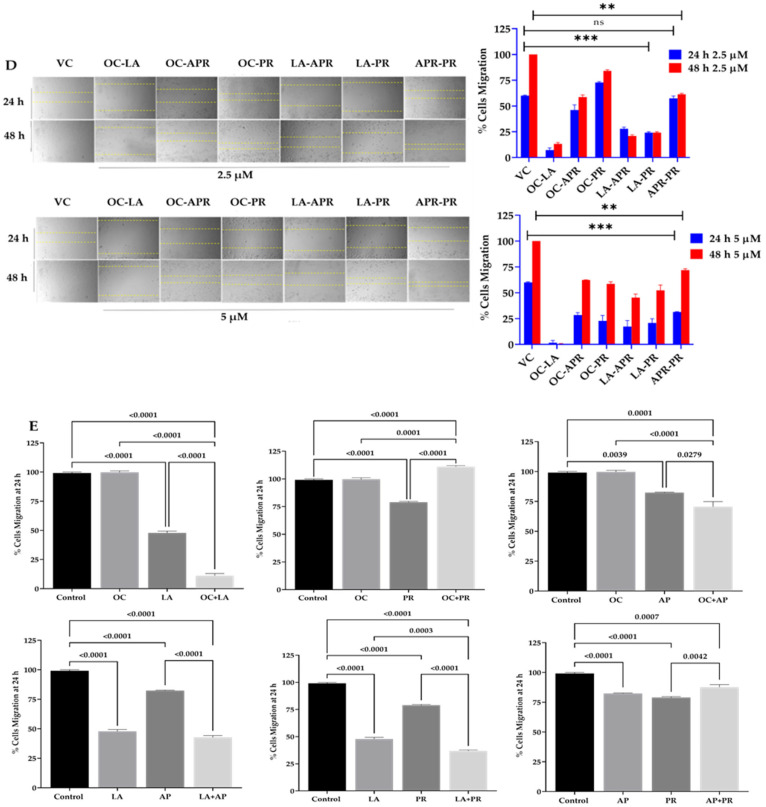
TNBC migration suppressive effects of olive phenolics using wound-healing scratch assay. (**A**) Antimigratory effects of nine EVOO phenolics at a single 10 µM treatment concentration against the TNBC MDA-MB-231 cells over 24 h and 60 h time points. (**B**) Quantitative antimigratory activity of EVOO phenolics against TNBC MDA-MB-231 cells over 24 h and 60 h time points. (**C**) Comparative time-dependent antimigratory concentration responses of the four most active EVOO phenolics APR, PR, LA, and OC against MDA-MB-231 cells over 24 and 48 h. (**D**) Discovery of the most antimigratory active combinations of the EVOO phenolics APR, PR, LA, and OC on MDA-MB-231 cells at 2.5 µM and 5 µM treatment concentrations over 24 h and 48 h. Dotted yellow lines in (**C**,**D**) represent the measured wound margins at the time of migration end point reading. (**E**) Quantitative antimigratory comparison of APR, PR, LA, and OC individual and combination therapies against MDA-MB-231 cells over 24 h treatment period was conducted using One-way ANOVA, followed by a post hoc Dunnett’s multiple comparisons test. ns: Non-statistical significance at *p* > 0.05. * Statistical significance at *p* < 0.05, ** statistical significance at *p* < 0.01, and *** statistical significance at *p* < 0.001.

Screening of a small library of EVOO phenolics (nine compounds) at a single dose of 10 µM over a period of 48 h on cells’ viability of the BC cell lines ZR-75-1 and MDA-MB-231 using the MTT assay identified OC, LA, and HOC (Table 1) as the most active hits. About 80.30% and 95.84% of MDA-MB-231 and ZR-75-1 cells, respectively, remained viable when treated with 10 µM OC over 48 h period (Table 1). About 63.43% and 38.74% of MDA-MB-231 and ZR-75-1 cells, respectively, remained viable with 10 µM LA over 48 h (Table 1). Similarly, 55.85% and 85.17% of MDA-MB-231 and ZR-75-1 cells, respectively, remained viable with 10 µM HOC (Table 1). A wound-healing scratch assay using the MDA-MB-231 cells was applied to assess the antimigratory effects of the nine olive phenolics at the same single 10 µM dose over a period of 24 h and an extended period of 60 h. Results demonstrated that OC, LA, (+)-1-acetoxypinoresinol (APR), and (+)-pinoresinol (PR) had the most potent antimigratory effect against the MDA-MB-231 cells (Figure 2A,B). An additional antimigratory concentration response assessment showed the potency of the most active four olive phenolics, APR, PR, LA, and OC, in a dose-dependent manner. Results showed marked antimigratory activity upon the exposure of the MDA-MB-231 cells to increasing OC, LA, APR, and PR doses (2.5, 5, and 7.5 µM) over 24 h and 60 h. LA and OC showed the best antimigratory effects in comparison to APR and PR (Figure 2C). The study used the TNBC MDA-MB-231 cells for the migration assay since it is a highly migratory aggressive and metastatic cell line, unlike the non-invasive ZR-75-1 BC cells.

### 2.3. LA and OC Combination Synergistically Inhibits Proliferation and Migration of MDA-MB-231 Cells

A wound-healing assay was performed using the MDA-MB-231 cells to assess the antimigratory effects of the four most potent EVOO phenolics as a combination matrix at 2.5 and 5 µM over 24 h and 48 h periods. Results identified combined LA-OC treatments as having the best antimigratory effect against the MDA-MB-231 cells migration (Figure 2D). Another wound-healing assay was performed to compare the effect of each individual olive phenolic with its corresponding combinations. Results showed an impressive synergistic effect for the combined LA and OC treatments in comparison to their individual treatments (Figure 2E).

### 2.4. Effects of OC and LA Individual and Combined Treatments on the Viability of BC Cell Lines

**Figure 3:**
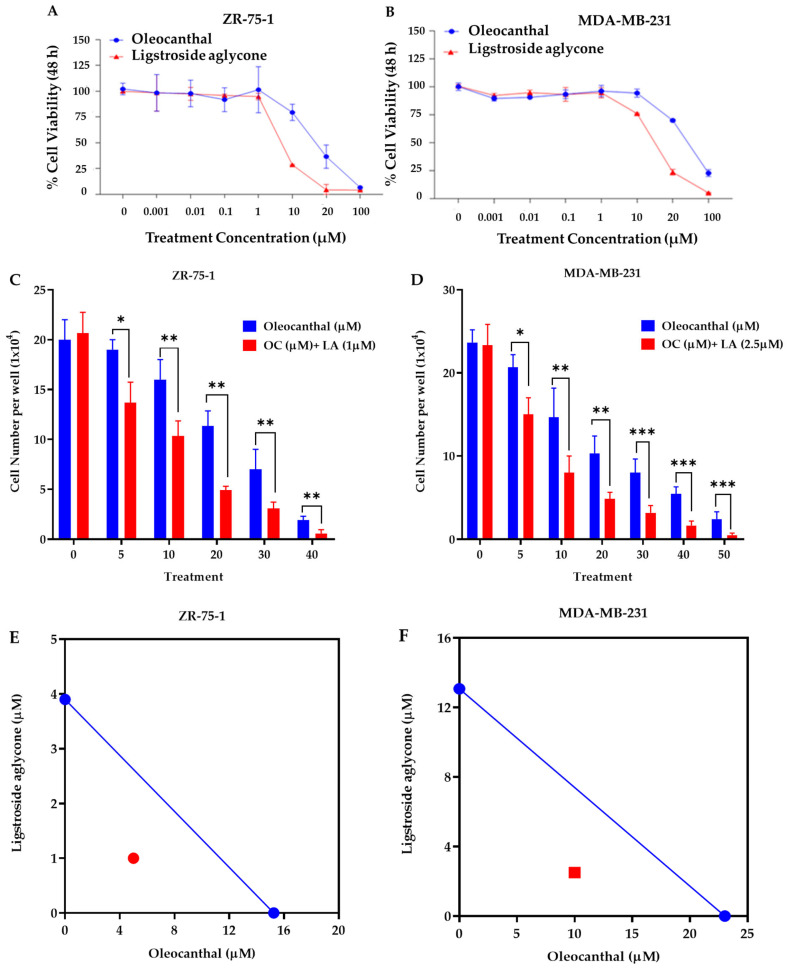
Effects of EVOO phenolics on in vitro BC viability. MTT assay concentration response curve for OC and LA against the viability of the luminal A ZR-75-1 cells (**A**) and the TNBC MDA-MB-231 cells (**B**). (**C**) Comparison of the effects of variable OC concentrations individually and combined with 1 µM LA on the viability of ZR-75-1 cells over 48 h treatment period. (**D**) Comparison of the effects of variable OC concentrations individually and combined with 2.5 µM LA on the viability of MDA-MB-231 cells over 48 h treatment period. (**E**) Isobologram of combined OC and 1 µM LA presenting effects on the viability of ZR-75-1 cells. (**F**) Isobologram of combined OC and 2.5 µM LA presenting effects on the viability of MDA-MB-231 cells. IC_50_ concentrations for OC and LA were plotted on the x-axis and y-axis, respectively, (blue dots). The solid line connecting these points represents the concentration of each compound required to induce the same relative growth inhibition when used in combination if the interaction between the compounds is additive. The data points on each isobologram represent the actual concentrations of OC and LA that induced 50% inhibition of cells growth when used in combination. Vertical bars indicate the percentage of colony formation relative to VC. Data analysis for treatments and control group values were determined using ordinary One-way ANOVA, followed by the post hoc Dunnett’s multiple comparisons test. * *p* < 0.05, ** *p* < 0.01 and *** *p* < 0.001 indicate statistical significance compared to their respective VC.

The effects of OC and LA on the viability of the luminal A ZR-75-1 BC cell line and the TNBC MDA-MB-231 cells were assessed using MTT assay. OC and LA concentrations ranging from 0.001–100 µM reduced ZR-75-1 cells viability in a dose-dependent manner, recording IC_50_ values of 15.3 µM and 3.9 µM, respectively. Furthermore, the same concentration range of OC and LA showed a reduction in cells viability of the MDA-MB-231 cells in a dose-dependent manner, recording IC_50_ values of 23.0 µM and 13.1 µM, respectively (Figure 3A,B). Combined treatment of 1 μM LA, a selected subeffective dose, with 2–40 μM OC significantly inhibited ZR-75-1 cells growth compared to both vehicle-treated control cells and the respective OC-treated cells. Similarly, a combination of a subeffective dose of 2.5 µM LA with 5–50 µM OC suppressed MDA-MB-231 cells growth (Figure 3C,D). Isobologram analysis for the effect of combination treatment of LA and OC in both BC cell lines indicated a synergistic inhibition of cells growth as the data points were located below the line of additivity in the isobolograms (Figure 3E,F). The IC_50_ value calculated for OC treatment in combination with LA was 5 μM against ZR-75-1 cells and 10 μM against MDA-MB-231 cells. The combination index (CI) is a quantitative representation of pharmacological interaction between two ingredients/entities [28,29,38]. CI values of <1, 1, and >1 indicate synergistic, additive, and antagonistic effects, respectively [28,29,38]. The CI value for the OC-LA combination was calculated using the following formula: CI = [OCc/O + LAc/L]. The O and L stand for the individual IC_50_ concentrations of OC and LA, respectively, that induced 50% cells growth suppression; OCc and LAc are the concentrations of OC and LA, respectively, that inhibited 50% of cells growth when used in combination (red circle in Figure 3E and red square in Figure 3F).

The calculated CI for proliferation-suppressive effects of combined LA and OC treatments indicated a high level of synergism. The CIs recorded for OC-LA combination against ZR-75-1 and MDA-MB-231 cells were 0.58 and 0.63, respectively. The synergistic interaction between OC and LA enhanced cancer cells sensitivity to OC treatment, evidenced by a multi-fold reduction in OC concentration needed to achieve the same BC proliferation suppression when combined with LA. The dose reduction index (DRI) for OC against ZR-75-1 cells is 3.1. DRI for OC against MDA-MB-231 is 2.3 [28,29,38]. The DRI for LA against ZR-75-1 cells is 3.9 and against MDA-MB-231 cells is 5.2. DRI values for each of OC and LA were calculated as follows: DRI OC = O/Occ, LA = L/LAc. DRI value is the fold-decrease in the dose for an individual compound when used in combination versus its concentration as a single agent needed to achieve the same effect level [28,29,38].

### 2.5. Comparison of the LA and OC Antiproliferative Activity Versus the Chemotherapy and Targeted Therapies

The antiproliferative activities of LA and OC against BC cells were compared with a diverse panel of chemotherapeutic and targeted therapeutic drugs, including the microtubule depolymerization disruptor paclitaxel (PTX), the first-generation EGFR inhibitor gefitinib (GFT), the selective estrogen receptor modulator tamoxifen (TAM), the estrogen receptor degrader fulvestrant (FUL), in addition to the standard selective SMYD2 inhibitor BAY-598. The MTT assay was used over 48 h treatment periods. Results showed that the taxane PTX was the most potent against both BC cell lines, with an IC_50_ in the low nM range. GFT, TAM, and FUL were far less potent with IC_50_ values in the low µM range with the luminal A ZR-75-1 BC cells. LA showed better activity against these cells than GFT and TAM and was comparable to FUL activity (Table 2). OC was much less active against ZR-75-1 cells. Similarly, TAM and FUL were not active against the TNBC as it lacks ER expression. GFT was marginally active against the TNBC cells MDA-MB-231, unlike PTX, which was superiorly potent (Table 2). LA was more active than GFT against TNBC, but OC was less potent. OC and LA antiproliferative activities were better than the standard selective SMYD2 inhibitor BAY-598 against both BC cell lines (Table 2).

### 2.6. Validation of the Antimigratory and Anti-Invasive Effects of LA and OC Individual and Combination Treatments Against the TNBC MDA-MB-231 Cells

**Figure 4:**
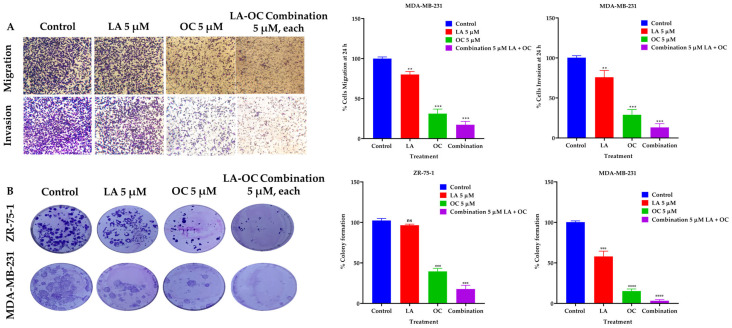
BC migration, invasion, and clonogenic growth-suppressive effects of OC and LA individual treatments and their combination. (**A**) Comparison of the LA and OC individual and combined treatment effects versus VC against the migration and invasion of MDA-MB-231 cells over 24 h treatment period. (**B**) The effects of individual 5 µM quantities of each of LA and OC and their combination treatments versus VC on the clonogenicity of ZR-75-1 and MDA-MB-231 BC cells. Vertical bars indicate the percentage of colony formation relative to VC. Data analysis of treatments and VC groups were determined using ordinary One-way ANOVA, followed by the post hoc Dunnett’s multiple comparisons test. ns = non-significant, ** *p* < 0.01, *** *p* < 0.001, and **** *p* < 0.0001 indicate statistical significance versus their respective VC.

Following the discovery of the LA and OC antimigratory activities against TNBC using wound-healing scratch assay, the Radius™ migration kit was used to further validate this activity. An individual treatment concentration of 5 µM of each of LA and OC, in addition to a combined 5 µM of each of LA, OC, was used to assess the antimigratory activity against MDA-MB-231 cells (Figure 4A). The 5 µM concentration is a subtoxic concentration since the IC_50_ for each of OC and LA is 23.0 and 13.1 µM, respectively, against MDA-MB-231 cells (Table 2). This assured the observed migration suppressive effects are not due to a cytotoxic effect but rather a pharmacological effect on tumor cells motility.

The BD Biocoat Matrigel invasion chamber, containing an 8 µm pore size PET membrane with a thin-layer Matrigel basement membrane matrix, was used to assess the anti-invasive effects of 5 µM of each of LA and OC, in addition to a combined 5 µM of OC and LA against MDA-MB-231 cells (Figure 4A). The LA and OC combination treatment showed a significant inhibition of MDA-MB-231 cells invasion capacity over the 24 h treatment period compared to individual 5 µM quantities of each of LA-, OC-, and VC-treated cells. OC individual treatment showed much better anti-invasive activity than individual LA treatment. The number of invaded cells in each well was counted using the ImageJ software (version 1.54k, NIH, USA). Each experiment was performed in triplicate for reproducibility and statistical relevance confirmation.

### 2.7. LA and OC Combination Treatments Inhibited the Clonogenicity of Invasive Breast Carcinoma Cells

A combination of LA and OC in a 5 µM concentration synergistically suppressed the clonogenicity of the luminal A BC cells ZR-75-1 and the TNBC MDA-MB-231 cells compared to individual treatments of each of LA and OC at the same concentration and the VC (Figure 4B). OC individual treatment showed much better colony formation-suppressive activity than LA individual treatment.

### 2.8. Effect of OC and LA Individual and Combination Treatments on the Progression of the Luminal a BC ZR-75-1 Cells Xenografted into Female Nude Mice

**Figure 5:**
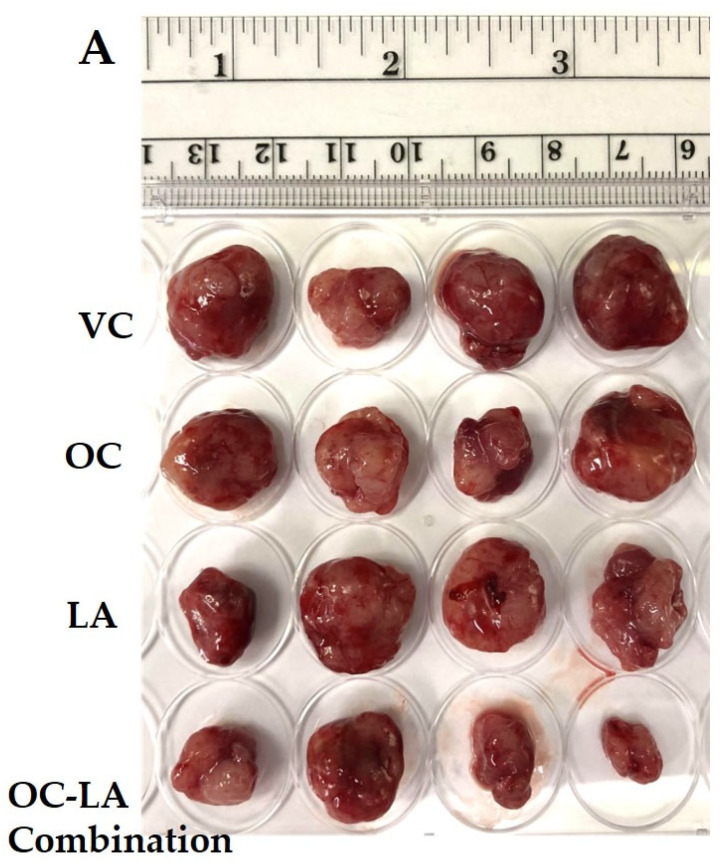

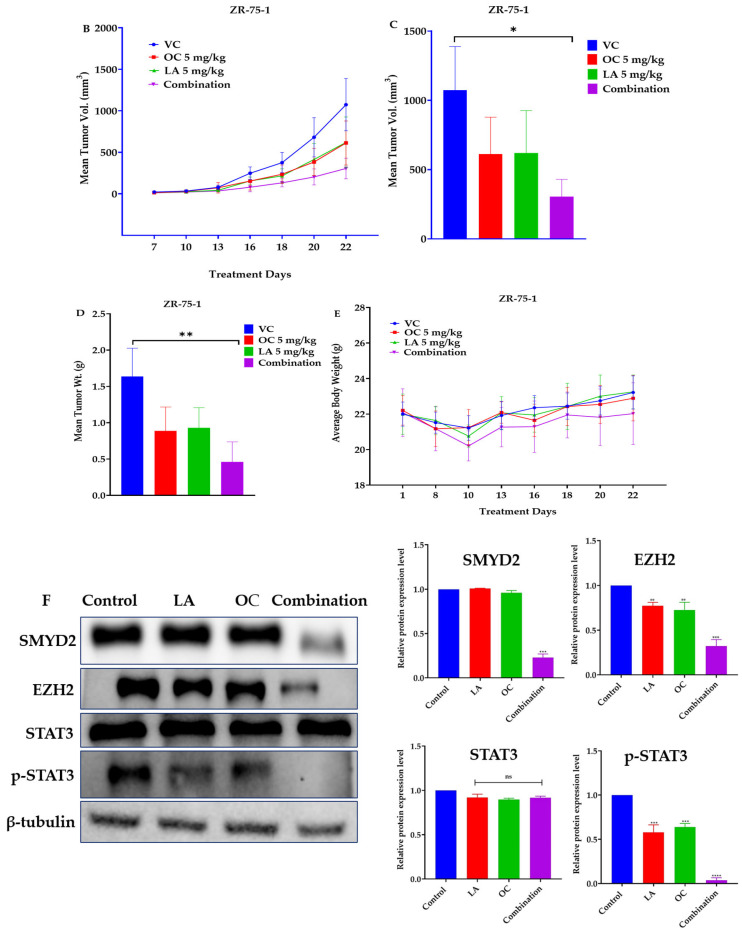
The in vivo ZR-75-1 BC progression suppressive effects of LA-OC combination (5 mg/kg, ip 3X/week) versus VC versus LA and OC individual treatments (5 mg/kg, ip 3X/week) in a nude mouse xenograft model. (**A**) Photographic comparison of surgically excised tumors in VC, individual LA, OC, and LA-OC combination treatments. (**B**) Comparative monitoring of tumor volume in LA-OC combination versus VC versus LA and OC individual treatments over 3 weeks. (**C**) Tumor volume on the last day of the study. (**D**) Comparison of LA-OC combination versus VC versus LA and OC individual treatments tumor weights. Results were presented as mean ± standard error of the mean (SEM). (**E**) Mouse body weight monitoring throughout the whole experiment course. (**F**) Western blots (**left panel**) and densitometric analysis for SMYD2, EZH2, STAT3, and p-STAT3 expression levels in VC, LA, and OC individual and combination treatments in the collected ZR-75-1 primary tumors (**right panel**). β-tubulin was used as the loading control. Densitometric values were normalized to β-tubulin. Ordinary One-way ANOVA was used for data analysis, followed by Dunnett’s multiple comparison test. ns = non-significant * *p* < 0.05, ** *p* < 0.01, *** *p* < 0.001, **** *p* < 0.0001 indicate statistical significance compared to their respective VC. Vertical bar graphs indicate the normalized integrated optical density for bands visualized in each lane.

The in vivo luminal A BC suppressive effects of OC and LA individual and combined treatments were examined in an orthotopic xenograft model of ZR-75-1 cells in female nude mice. The mean tumor volume of the VC-treated mice at the study end was 1073.53 ± 148.4 mm^3^. The mean tumor volumes recorded for the individual treatments of each of OC and LA (5 mg/kg, 3X, ip) were 612.91 ± 82.45 mm^3^ and 619.85 ± 86.31 mm^3^, respectively, indicating a reduction in tumor volume by 42.9% and 42.2%, respectively, versus the VC group (Figure 5A–C). Interestingly, the combined OC-LA treatment, 5 mg/kg, ip, 3X/week each, showed the most prominent reduction in tumor volume compared to the VC and individual therapies. Tumor volume recorded for the OC-LA combination treatments was 305.61 ± 41.25 mm^3^, demonstrating a 71.5% reduction in ZR-75-1 tumor volume. The mean tumor weight for the VC-treated group at the study end was 1640 ± 190 mg, and the mean ZR-75-1 tumor weights recorded for the individual treatments of OC and LA (5 mg/kg, ip, 3X/week) were 890 ± 150 mg and 930 ± 130 mg, respectively, indicating a reduction in ZR-75-1 tumor weights by 45.7% and 43.3%, respectively, compared to the VC-treated group (Figure 5D). The co-administration of OC and LA at 5 mg/kg, ip, 3X/week, each, showed the most significant reduction in ZR-75-1 tumor weight compared to VC and OC, and LA individual treatments. The tumor weight recorded for the OC-LA combination treatment was 460 ± 140 mg, demonstrating a reduction in tumor weight by 72.0%. OC and LA individual and combined treatments did not show a significant reduction in mouse average body weight when compared to VC-treated animals throughout the study course, indicating the potential preliminary safety profile for the EVOO phenolics OC and LA treatments (Figure 5E).

Western blotting analysis of the collected ZR-75-1 cell tumors at the study end revealed a significant downregulation of the relative protein expression levels of the SMYD2 and its downstream effector EZH2 (Figure 5F). The activated/phosphorylated STAT3 expression level was significantly downregulated in the tumors collected from the combined OC-LA-treated group, unlike the individual OC-, LA-, and VC-treated animals (Figure 5F). Meanwhile, the total STAT3 expression was not affected in all groups (Figure 5F).

### 2.9. Immunofluorescence Expression Comparison of Ki-67 and Histopathological Effects of OC-LA Combination Treatment in Collected ZR-75-1 Primary Tumors

**Figure 6:**
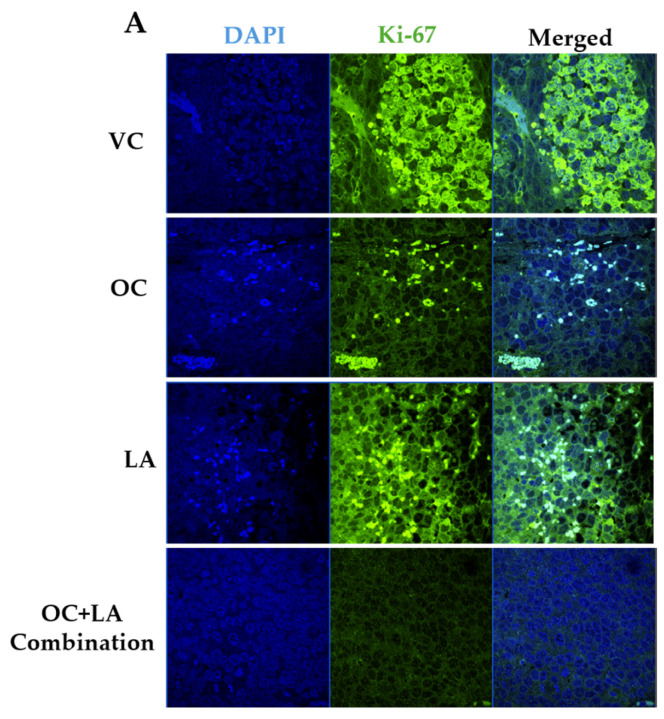

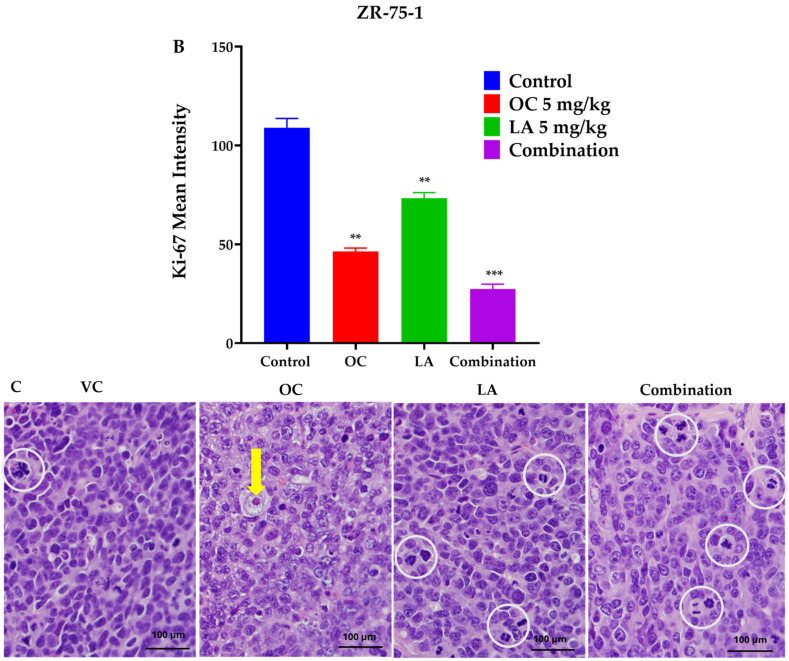
Immunofluorescence staining of total levels of Ki-67 and histopathological study in collected luminal A BC ZR-75-1 tumors. (**A**) Comparison of treatment effects on immunofluorescence of Ki-67 in ZR-75-1 tumors for VC, individual OC, LA, and OC-LA combined-treated groups. (**B**) Ki-67 expression level quantitation for individual OC and LA, their combination, and VC-treated groups. Blue staining represents nuclei, counterstained with DAPI. Green staining indicates the presence of the specific protein of interest, Ki-67. Immunofluorescence intensity staining was quantified in several optical fields as detailed in the Materials and Methods section. Student’s *t*-test used for statistical analysis. Ordinary One-way ANOVA was used for data analysis, followed by Dunnett’s multiple comparison test. ns = non-significant. ** *p* < 0.01, *** *p* < 0.001 indicate statistical significance compared to their respective VC. (**C**) Histopathological examination of H&E-stained ZR-75-1 tumor tissues of LA-OC combination versus VC and individual LA and OC treatments at 40× magnification and 100 µm scale bar. Yellow arrows refer to prominent nuclei. White circles refer to mitotic figures.

The immunofluorescence study explored the effects of OC-LA monotherapies and combination treatments on the tumor progression marker Ki-67. Results showed significant suppression of Ki-67 expression level in combined OC-LA-treated primary tumor sections, compared to individual OC, LA, and VC-treated tumors (Figure 6A,B). The effective suppression of Ki-67 expression by the synergestic OC-LA combination clearly justifies its effective antiproliferative activity in BC. H&E-stained microscopic sections in collected tumors at the study end for VC, individual OC, LA, and combined OC-LA treatments exhibited higher nuclear to cytoplasmic ratios, occasional prominent nucleoli, and mitotic figures (Figure 6C). All tumors exhibited at least focal areas of necrosis and fibrosis. The areas of fibrosis in the OC-treated group were larger than those of the VC and LA groups. The combined OC-LA-treated group exhibited the most fibrosis in the examined sections (Figure 6C).

### 2.10. Effect of Individual OC and LA and Their Combination Treatments on the Metastasis of the TNBC MDA-MB-231-Luc Cells in a Nude Mouse Tail Vein Model

**Figure 7:**
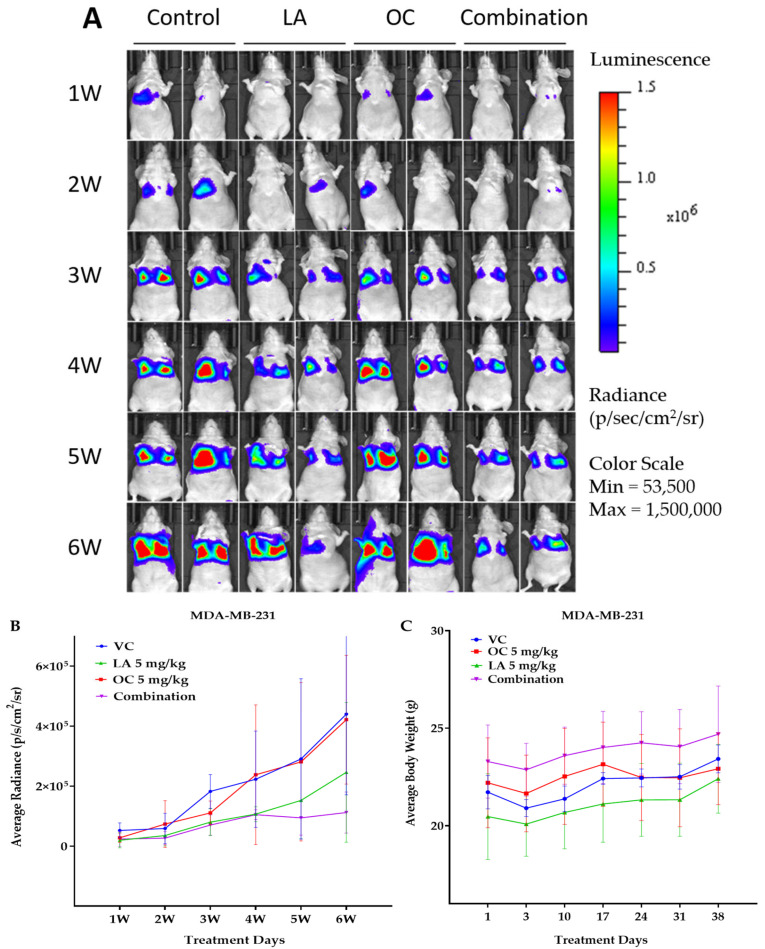

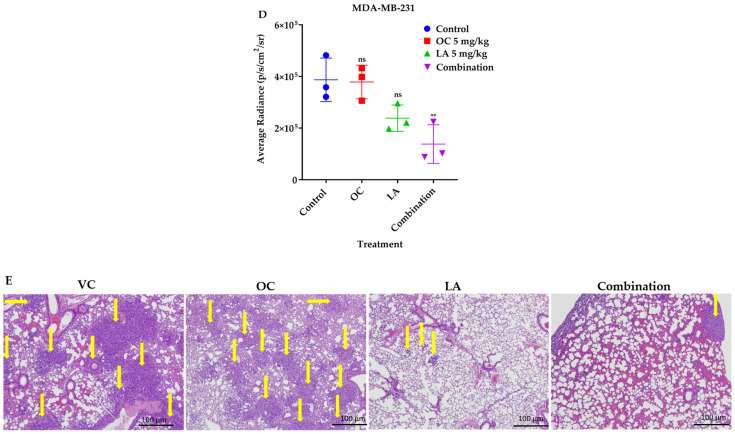
The in vivo VC, individual 5 mg/kg each of OC and LA, along with the LA-OC, 5 mg/kg each, combination against the MDA-MB-231-Luc cells metastasis in a tail vein female nude mouse model. (**A**) Weekly live animal bioluminescence monitoring of MDA-MB-231-Luc cells tumor progression and metastasis. (**B**) Tumor bioluminescence intensity quantitative monitoring in live animals. (**C**) Average body weight monitoring of study mice groups over experiment course. (**D**) Post-sacrifice quantification of the tumor bioluminescence intensity in collected animal lungs. Ordinary One-way ANOVA was used for data analysis, followed by Dunnett’s multiple comparison test. ns = non-significant. ** *p* < 0.01 indicates statistical significance compared to their respective VC. (**E**) Histopathological comparative examination of H&E-stained lung tissues of VC, individual LA, OC, and LA-OC combination treatments at 4× magnification and 100 µm scale bar. Yellow arrows refer to metastatic lesions.

Since OC and LA individual and combination treatments showed good in vitro antimigratory, anti-invasive, and clonogenicity suppressive effects against TNBC, their in vivo antimetastatic effects were assessed using the TNBC MDA-MB-231-Luc cells in the female nude mouse tail vein model. Biweekly live animal bioluminescence imaging was used to monitor TNBC progression and metastasis (Figure 7A). Bioluminescence imaging showed notable suppressive effects for the OC-LA combination on the TNBC progression and metastasis over the study period (Figure 7A,B). Mouse body weight monitored over the study course showed no significant changes observed for the individual and OC-LA combination treatments versus the VC group (Figure 7C).

The quantification of bioluminescence tumor intensity in collected animal lungs at the study end revealed effective tumor bioluminescence reduction in lungs of mice treated with the LA-OC combination compared to VC and the individual LA and OC treatments. This clearly indicated the effective reduction of the TNBC clonogenicity at the treated animals’ lung tissues, suggesting the potential of phenolics-rich EVOO to limit TNBC metastasis to the lung (Figure 7D). Histopathological investigations of treated mouse lungs demonstrated numerous tumor cells metastatic foci in subpleural, peribronchial, and perivascular locations, and in interalveolar septae in the VC lung tissues (Figure 7E). Since OC individual treatment was used at a subeffective 5 mg/kg dose, the OC-treated group showed abundant metastases and tumor in subpleural, peribronchial, and perivascular locations and along interalveolar septae. The 5 mg/kg LA, ip, 3X/week-treated mice revealed fewer metastatic foci than the VC group in histopathological examination of collected animal lung sections (Figure 7E). Meanwhile, the LA-OC co-treated group had many fewer lung metastatic foci than the individual OC, LA, and VC-treated groups (Figure 7E).

### 2.11. Immunofluorescence Expression Comparison of Ki-67 and CD31 in Collected MDA-MB-231 Lung Tissues

**Figure 8:**
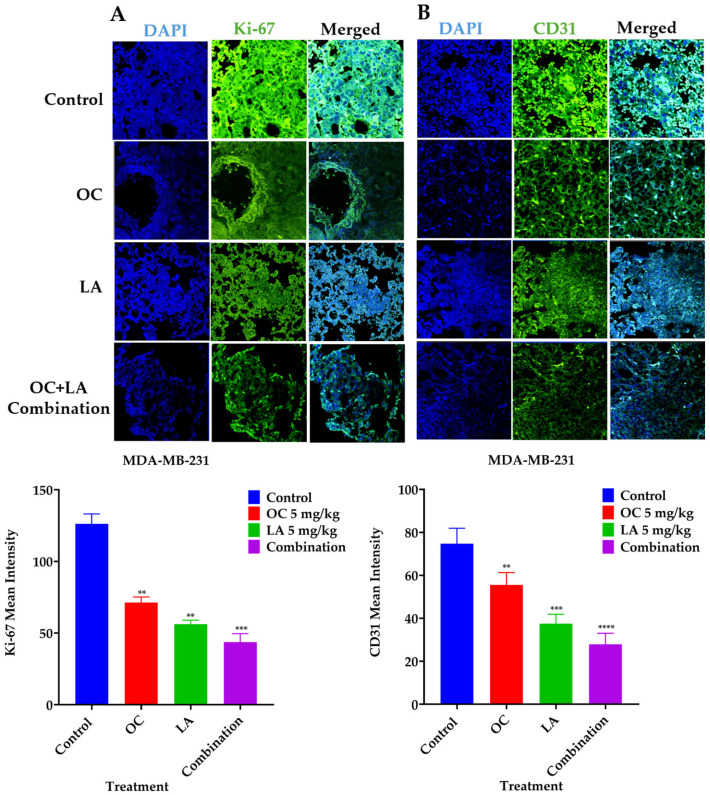
Immunofluorescence staining of total levels of Ki-67 and CD31 in collected TNBC MDA-MB-231-Luc lung tissues. (**A**) Comparison of VC, individual LA and OC, as well as LA-OC combination treatment effects on the immunofluorescence of the proliferation marker Ki-67 in MDA-MB-231-Luc tumors metastatic foci in animal lung sections. (**B**) Comparison of VC, individual LA and OC, as well as LA-OC combination treatment effects on the immunofluorescence of the vasculogenesis marker CD31 in MDA-MB-231-Luc tumors metastatic foci in animal lung sections. Ordinary One-way ANOVA was used for data analysis, followed by Dunnett’s multiple comparison test. ** *p* < 0.01, *** *p* < 0.001, **** *p* < 0.0001 indicate statistical significance compared to their respective VC. Blue staining represents nuclei, counterstained with DAPI. Green staining indicates the presence of the specific proteins of interest, Ki-67 and CD31. Immunofluorescence intensity staining was quantified in several optical fields as detailed in the Materials and Methods section.

Immunofluorescence study comparing the effects of individual LA, OC, and OC-LA combination treatments versus VC on the tumor progression marker Ki-67 expression and the vascular endothelial vasculogenesis marker CD31. Results showed significant suppression of both Ki-67 and CD31 expression levels in OC-LA combination-treated versus individual LA, OC, and VC-treated lung tumors (Figure 8A,B).

## 3. Discussion

Therapeutic approaches targeting luminal A BC and TNBC face distinct challenges due to their divergent molecular profiles [2,3]. Luminal A tumors often develop resistance to hormonal therapies over time, limiting the long-term efficacy of targeted therapies. In contrast, TNBC lacks expression of estrogen, progesterone, and HER2 receptors, eliminating the targeted therapies option and leaving chemotherapy as the main intervention option [4]. Chemotherapies have high toxicities, develop rapid resistance, and sometimes have a limited therapeutic response [4]. Furthermore, TNBC is highly heterogeneous, complicating intervention drug development; hence, discovery of new alternative therapeutics addressing these challenges is a high priority.

Data acquired from the TCGA database showed the overexpression of SMYD2, STAT3, and EZH2 among BC subtypes, highlighting their potential contributions to BC pathogenesis. Their consistent upregulation in luminal and HER2^+^ BC, TNBC, as well as in other cancers, highlights their relevance as explicit oncogenic drivers. SMYD2 and EZH2 showed the most significant dysregulation, indicating their particular importance for BC progression. Screening of a small library of nine EVOO phenolics identified OC, LA, and HOC with moderate cytotoxic effects against BC. Several BC phenotypes also tend to invade, migrate, and metastasize to the surrounding and distant sites [39]. Thus, limiting BC motility is an utmost therapeutic goal. Combination studies for OC and LA reduced BC cells viability in a dose-dependent manner. Individually, LA showed better BC cells viability-suppressing potency versus OC. OC and LA showed a strong synergistic effect against both ZR-75-1 and MDA-MB-231 cells viability. A CI value of <1 indicated a synergistic effect, and a DRI value >1 validated the enhanced anti-BC efficacy at much reduced OC-LA doses. DRI values > 1 are advantageous because this demonstrates that the compound achieves therapeutic efficacy at much lower individual compound concentrations, reducing the risk for potential off-target effects. Identification of synergistic activity of EVOO phenolics at lower concentrations can translationally be applied to select the OC-LA-rich EVOO varieties for use by BC patients and survivors.

Comparison of the LA and OC antiproliferative activity against a panel of chemo- and targeted therapies shows that, although PTX demonstrated superior activity on BC cells viability compared to OC in the MTT assay, OC remains a promising entity for invasive BC, either alone or combined with other entities, due to its superior selectivity to malignant cells versus non-tumorigenic cells, potent activities against cancer motility-invasion assays, favorable safety profile, absence of neuropathic side effects unlike taxanes, and high in vivo oral anti-BC potency. The combination of LA and OC at 5 µM, which is a subtoxic concentration, significantly inhibited MDA-MB-231 cells migration and invasion, confirming their pharmacological antimigratory and anti-invasive effects. The 5 µM treatment concentration for each of LA and OC is a physiologically relevant concentration that can mimic the low natural abundance of both EVOO phenolics in the commercial quality EVOO brands. LA and OC combination treatments showed a significant inhibition for the MDA-MB-231 cells migration over the 24 h treatment period compared to individual 5 µM of each of LA, OC, and VC-treated cells.

Clonogenicity assays showed a synergistic effect for the LA-OC combination against both ZR-75-1 and MDA-MB-231 BC cell lines. LA-OC combination treatment likely restrained tumor cells motility without causing direct cytotoxicity or affecting cells’ viability. The clonogenicity assay is based on the ability of a single tumor cell to adhere and form a subsequent viable colony. This in vitro experimental model resembles the in vivo distant recurrence/metastasis process in which the circulating tumor cell adheres to a distant preferential organ with a proper microenvironment, favoring and promoting this adherence (organotropism), forming a colony and developing subsequent metastatic foci [38]. The colony formation assay model is the in vitro mimic of the nude mouse tail vein-metastasis in vivo model used in this study.

The LA-OC combination synergistically reduced tumor volume and weight in the ZR-75-1 xenograft model, achieving a significant tumor progression suppression. Furthermore, no significant body weight changes were observed, suggesting a plausible favorable safety profile. These results support the potential of OC and LA in combination, as promising therapeutic nutraceutical candidates for luminal A BC. SMYD2-EZH2 were recently documented as molecular targets for OC in prostate and colorectal cancers [40]. Both lysine methyltransferases have been validated as critical molecular targets in many cancers, including BC [10,11,12,13,41,42]. STAT3 proved to be a SMYD2 downstream tyrosine kinase substrate and OC molecular target [10,11,12,26,43]. Western blot results clearly proved that the OC-LA combination was effective in synergistically reducing SMYD2-EZH2-p-STAT3 expression levels in the luminal A ZR-75-1 invasive BC. The lysine methyltransferase SMYD2 was reported to methylate EZH2 at lysine 307 (K307), which promotes BC cells proliferation, EMT, and invasion [42,43,44]. Moreover, EZH2 promotes BC by directly methylating and activating STAT3 as a post-transcriptional modification [13]. STAT3 activation by EZH2 is crucial for BC improved cells survival, proliferation, and migration [13]. Collectively, the combined EVOO phenolics OC-LA proved effective in suppressing luminal A BC progression by targeting the SMYD2–EZH2–STAT3 axis. The OC-LA combination notably suppressed Ki-67 expression, confirming its enhanced antiproliferative activity in BC. Histological examination revealed increased fibrosis and tumor necrosis in the combination-treated group, indicating enhanced therapeutic effects. These findings reinforce the synergistic efficacy of OC and LA in targeting tumor progression at both molecular and tissue levels.

OC was previously tested in female nude mouse xenograft and transgenic mouse models for BC progression and recurrence, but never tested for antimetastatic activity [27,28,29]. The in vivo antimetastatic effect of the LA-OC combination was assessed in a female nude mouse tail vein model using TNBC MDA-MB-231-Luc cells. Results revealed a remarkable controlled cancer dissemination exerted by LA-OC combination treatments. Inevitably, this model should show the highest tumor cells clonogenicity in the animal lungs, since the iv-injected tumor cells will circulate within the blood stream to reach the lungs, which offer favorable tumor microenvironment [45]. Thus, this model is an ideal mimic for in vivo clonogenicity. The regular-standard in vivo OC therapeutic dose in nude mouse models is usually 10 mg/kg [27,28,29,31,40]. The selection of the low 5 mg/kg subeffective dose in this study was intended to mimic the low natural occurrence of these phenolics in EVOO. Translationally, the human equivalent dose (HED) for the 5 mg/kg used in this study in mice can be calculated as follows: HED mg/kg = Animal dose (mg/kg) x K_m_ ratio = 0.41 mg/kg in humans [46]. The correction factor K_m_ is the ratio of the average body weight (kg) to body surface area (m^2^). K_m_ is constant for each species. The K_m_ value for mice is 3 and for humans is 37 [46]. Thus, a daily intake of 0.41 mg/kg OC by a human with an average body weight of 70 kg will translate to a total of 28.7 mg, which can match the used dose in this study. This dose is physiologically realistic, considering the use of a daily intake of 28.7 mL phenolics-rich EVOO, which can normally contain up to 1000 mg OC/L. Microenvironments that interface tumor cells and specific organs play a vital role in organ-specific metastasis. Circulating tumor cells travel to distant organs, where they initiate new tumors when the microenvironment is favorable for subsequent clonogenicity [45,47,48]. The LA-OC combination markedly suppressed lung metastases, highlighting its potential to limit BC spread. Immunofluorescence confirmed significant inhibition of Ki-67 and CD31, indicating reduced proliferation and endothelial vasculogenesis. These findings support the combination’s anti-metastatic and anti-angiogenic efficacy in vivo.

OC has never been tested in clinical trials as a pure entity [49]. The 13 hits for OC on Clinicaltrials.gov show OC-rich EVOO or combination testing [49]. OC-rich EVOO was tested in chronic lymphocytic leukemia by Rojas 2022, NCT04215367 [50]. This study did not use pure OC but used EVOO-rich in OC [50]. Patients used for 9 months daily oral 40 mL EVOO containing either 416 mg/L OC + 284 mg/L HOC or 82 mg/L OC + 33 mg/L HOC [50]. This translates to the highest patient total OC daily intake of 16.6 mg, which translates to 0.24 mg/kg for a 70 kg average body weight patient. A study conduced in Greece (NCT04520126) used a 3:1:2 OC–hydroxytyrosol–oleuropein combination for cardiovascular–endothelial function [49]. Other clinical trials reported the use of EVOO-nutraceutical combinations for metabolic, cardiovascular, gut–brain axis, multiple sclerosis, neurofibromatosis, or cognitive diseases [49]. This study’s results and literature studies indicate the near-future clinical promise for OC and LA for testing to control BC in humans. The main translational limitation of EVOO use as a source for useful anti-BC nutraceuticals is the erratic natural occurrence of various EVOO phenolics, including OC and LA. Their natural occurrence in EVOO is based on diverse factors, including genetic origin, environment, collection time, handling, malaxation, and several other factors. Standardized EVOO with known specific OC and LA levels can address this challenge and facilitate patients selection of the best EVOO varieties for prospective nutraceutical use.

## 4. Materials and Methods

### 4.1. Chemicals and Reagents

All chemicals and reagents were purchased from Sigma-Aldrich (St. Louis, MO, USA), unless specified differently. Organic solvents were obtained from VWR (Suwanee, GA, USA), dried by standard procedures, packaged under nitrogen in Sure/Seal bottles, and stored over 4 Å molecular sieves, unless otherwise indicated. Antibodies were purchased from Cell Signaling Technology (Beverly, MA, USA), except where noted. Antibodies typically used at 1:1000 dilution unless otherwise noted. Cell culture reagents were purchased from Life Sciences (Carlsbad, CA, USA).

### 4.2. Extraction of Olive Phenolics from Extra-Virgin Olive Oil

The EVOO phenolics *S*-(−)-Oleocanthal (OC), *S*-(−)-hydroxyoleocanthal (HOC), tyrosol (TY), hydroxytyrosol (HT), *S*-ligstroside aglycone (LA), *S*-(−)-oleuropein aglycone (OA), (+)-pinoresinol (PR), and (+)-1-acetoxypinoresinol (APR) were isolated from Greek EVOO collected batches in the fall of 2020 and fall of 2021 (The Governor, Corfu, Greece). Separation of EVOO phenolic compounds was performed using an in-house-developed novel liquid–liquid extraction methodology, partitioning EVOO with deionized water, entrapping phenolics on Sorbtech, Sepabeads Resin Styrenic Adsorbent Sp-70-01 entrapment resin (Sorbent Technologies, Norcross, GA, USA), eluting phenolics with acetone, followed by size exclusion chromatography of dried acetone extract on Sephadex LH20, using CH_2_Cl_2_-EtOAc careful gradient elution [30]. The identity and purity of olive phenolics were validated by q^1^H NMR analysis using a JEOL JNM-ECZL400S FT-NMR system with Delta™ processing software (version 6.3, Peabody, MA, USA) and high-resolution mass spectrometric analysis using a JEOL JMS-T100LP AccuTOF LC-plus instrument (Peabody, MA, USA), confirming >99% purity for each tested olive phenolic [30]. Spectroscopic identity was based on extensive 1D and 2D NMR analyses and comparison with literature values, especially the chemical shift of LA protons H-3 (δ 7.59, singlet) and H-8 (δ 9.57, broad singlet) [22,30]. The quantitation of OC purity was based on the integration ratio of the OC key H-3 aldehydic proton signal at δ 9.23 and the residual CHCl_3_ peak in the CDCl_3_ at δ 7.24 [30]. The 99% purity EVOO phenolic samples were stored frozen in amber glass vials under N_2_ gas [30]. *S*-(−)-oleuropein was extracted from commercial olive leaf extract capsules, 50% standardized oleuropein content (Nusapure^®^, Amazon, Seattle, WA, USA), extracted with MeOH, and the remaining residue after defatting with *n*-hexanes was size-exclusion-chromatographed on Sephadex LH20, using CH_2_Cl_2_-MeOH gradient elution [48].

### 4.3. Cell Lines and Culture Conditions

Human BC cell line ZR-75-1 (ER-positive luminal A) was obtained from Charles River Laboratories, Inc. (Wilmington, MA, USA), while the human luciferase-tagged TNBC cell line MDA-MB-231-Luc was purchased from GenTarget Inc. (San Diego, CA, USA). Cells were cultured in Roswell Park Memorial Institute (RPMI-1640) provided with 10% fetal bovine serum (FBS), penicillin G (100 U/mL), streptomycin (100 ng/mL), and sodium pyruvate (100 ng/mL). All cells were maintained in a humidified incubator at 37 °C with 5% CO_2_. For sub-culturing, cells were washed with Ca^2+^- and Mg^2+^-free phosphate-buffered saline (PBS) and incubated in 0.05% trypsin containing 0.02% Ethylenediaminetetraacetic acid (EDTA) in PBS for 5–15 min at 37 °C.

### 4.4. Tumor Cells Proliferation Assay

MDA-MB-231 and ZR-75-1 cells were plated in 96-well plates at a density of 1 × 10^4^ cells per well and incubated overnight at 37 °C in a 5% CO_2_ humidified incubator for attachment. The next day, treatment working solutions were prepared in DMSO, and multiple different concentrations of treatments were added to the wells in triplicate, then cells were incubated at 37 °C for 48 h. Media were gently aspirated. About 100 μL of fresh media and 100 μL of MTT solution were added to each well, and cells were incubated for an additional 3 h. Media were carefully removed, and formazan crystals were dissolved in DMSO. Optical density was measured at 570 nm using a Synergy 2 microplate reader (BioTek, 242 Winooski, VT, USA). Cells number deduced from a standard curve performed at the beginning of each experiment. The IC_50_ values were calculated using GraphPad Prism version 8.01 (GraphPad Software, San Diego, CA, USA). Experiments were conducted in triplicate to confirm reproducibility and statistical significance.

### 4.5. Wound-Healing Scratch Assay

MDA-MB-231 cells were seeded in 24-well plates at a density of 5 × 10^3^ cells/well and incubated overnight for attachment at 37 °C in a 5% CO_2_ humidified incubator. Wounds were then scratched using sterile 200 μL pipette tips. Cells were repeatedly rinsed with PBS and re-incubated in 1% serum media containing different treatment concentrations from a 10 mM stock solution in DMSO and DMSO as VC. Wound images were captured at zero time and monitored for wound closure up to 24 h. When wounds were about to close, the media were removed, and cells were washed with cold PBS and fixed with ice-cold MeOH, stained with Giemsa stain. Finally, wound images were taken for treatment and VC groups using a Nikon Ti2-A Inverted Intelligent microscope (Nikon Instruments Inc., Melville, NY, USA). The percentages of each treatment’s cell migration were calculated using the following formula: Percent cell migration = [T0 − Tt − Tdmso]/[T0 − Tdmso] × 100, where T0 is wound thickness at zero time, Tdmso is the wound thickness in DMSO-treated control wells, and Tt is the wound thickness in treatment wells. Experiments were performed in triplicate to ensure reproducibility and statistical significance.

### 4.6. Transwell Migration Assay

The Radius™ 96-Well Cell Migration Assay kit was used (Cell BioLabs, San Diego, CA, USA). MDA-MB-231 cells were cultured in each well’s upper chamber (5 × 10^5^ cells/well), containing either VC or individual phenolics (OC or LA) in a concentration of 5 µM each or combination of both treatments in a concentration of 5 µM. Aliquots of 10% FBS serum- media were supplemented to each lower chamber well as a chemoattractant. After 24 h, all chambers were washed with PBS and fixed with ice-cold MeOH for 10 min, then stained with Giemsa stain. Finally, chambers were photographed using an inverted Nikon microscope. Results were expressed as the percentage of migration and normalized to VC, which assumed 100% migration. Assays were executed in triplicate to ensure reproducibility and statistical significance.

### 4.7. Invasion Assay

The BD BioCoat™ BD Matrigel™ Invasion kit (BD Biosciences, San Diego, CA, USA) was used. MDA-MB-231 cells were seeded in each upper chamber (5 × 10^5^ cells/well) and precoated with Matrigel with either the VC or individual phenolics (OC or LA) in concentrations of 5 µM each or combination of both treatments in a concentration of 5 µM. Aliquots of 10% serum-containing media were added to the lower chambers as chemoattractant. After 24 h, all the chambers were rinsed with PBS and fixed with ice-cold MeOH for 10 min, then stained with Giemsa stain. Finally, an inverted Nikon microscope (Nikon Instruments Inc., Melville, NY, USA) was used to capture images of the chambers. Results were expressed as the percentage migration normalized to the VC, which assumed 100% migration. Experiments were performed in triplicate to ensure reproducibility and statistical significance.

### 4.8. Colony Formation Assay

ZR-75-1 and MDA-MB-231 cells were seeded in 12-well plates at a density of 1 × 10^3^ per well, incubated for 3 days till attachment and differentiation at 37 °C in a 5% CO_2_ humidified incubator. Cells were assigned into VC, individual phenolics (OC or LA) in concentrations of 5 µM each, and a combination of both treatments in a concentration of 5 µM in 1% serum media from a 10 mM stock solution in DMSO. Media with treatments were changed every other day for a 12-day incubation period. At the end of the experiment, media were aspirated, colonies washed with cold PBS, fixed with ice-cold MeOH, and stained with Giemsa stain. Images were captured using a digital camera, and colonies were manually counted. Experiments were repeated three times to ensure reproducibility and statistical significance.

### 4.9. Animal Models and Treatment Modes

Female athymic nude mice (Foxn^1nu^/Foxn^1+^, aged 5–6 weeks) were obtained from Envigo (Indianapolis, IN, USA). The mice were maintained at the University of Louisiana at Monroe (ULM) vivarium animal facility. Animals were housed in filter-top cages with Alpha-Dri bedding in a clean room environment, provided with free access to food and water. The cages were placed on ventilated racks equipped with high-efficiency particulate air (HEPA) filtration. Environmental conditions were controlled at 25 °C, 55–65% relative humidity, with a 12 h light/dark cycle. Animal experiments were approved by the Institutional Animal Care and Use Committee (IACUC), with the protocol number 19 NOV-KES-01. All experiments were performed in strict accordance–compliance with the NIH-guided good animal practices. Animal welfare and experimental protocols were strictly followed to minimize discomfort and distress to the mice throughout the study course.

#### 4.9.1. Orthotopic Nude Mice Xenograft Tumor Model

Approximately 5 × 10^6^ cells of the ER^+^ luminal A BC cell line ZR-75-1 suspended in Matrigel/RPMI-1640 were injected subcutaneously into the mouse mammary fat pad. Once the tumors became palpable, reaching an average 30–50 mm^3^ volume (nearly 14 days post xenografting), mice were randomly assigned into 4 groups, 4 mice per group: VC (sterile PBS), OC individual treatment group (5 mg/kg, ip, 3X/week), LA individual treatment group (5 mg/kg, ip, 3X/week), and a combination of both OC and LA (5 mg/kg, each, ip, 3X/week). Dosing continued over a period of 3 weeks. Tumor volume was measured using the standard formula: tumor volume (mm^3^) = [(length × width^2^)/2]. Animals were monitored for body weight changes and any signs of treatment- or vehicle-related toxicity. Animals were sacrificed at the end of the study, and tumors were collected and kept at −80 °C for subsequent analysis.

#### 4.9.2. Tail Vein Nude Mice Xenograft Tumor Model

The TNBC MDA-MB-231-Luc luciferase-labelled cells, approximately 1 × 10^6^ cells in sterile PBS, were xenografted intravenously into the tail veins of the female nude mice. Bioluminescence was measured by imaging 2% isoflurane anesthetized mice using an IVIS Lumina series III (Perkin Elmer, Waltham, MA, USA) imaging system after intraperitoneally (ip) injecting with D-luciferin (XenoLight D-luciferin K^+^ salt bioluminescent Substrate, PerkinElmer) at a dose of 75 mg/kg per animal in sterile PBS [44]. The photons emitted from luciferase-expressing cells within the animal body and transmitted through the tissue were quantified using the Living Image software program (PerkinElmer, Version 4.7.3, PerkinElmer, Waltham, MA, USA). Images representing bioluminescence intensity (blue, least intense, and red, most intense) were generated and quantified as photons/second. Mice were randomly assigned into 4 groups of 4 mice in each group: VC (sterile PBS), OC monotherapy treatment group (5 mg/kg), LA monotherapy treatment group (5 mg/kg), and a combination of both OC and LA (5 mg/kg, each). Dosing immediately started post xenografting intraperitoneally (ip), 3X/week for 6 weeks. Animals were imaged and bioluminescence was recorded once a week to monitor the tumor clonogenicity at each mouse’s lungs. The animals’ health was monitored routinely for clinical or behavioral changes, weight loss, or any signs of altered motor ability. At the study end, mice were sacrificed according to the approved IACUC protocol, and bioluminescence images of each mouse’s whole body and collected organs (lung, brain, liver, spleen, heart, and kidney) were captured, then immediately fixed in 10% neutral buffered formalin for 48 h. The tissues were further transferred to 70% ethanol for further analysis.

### 4.10. Immunohistochemistry (IHC) Study

The IHC slides, 5 μm thick sections acquired at AML Laboratories (Jacksonville, FL, USA), were made from paraffin-embedded tumor tissue samples of the ZR-75-1 xenograft tumor model and the MDA-MB-231-Luc metastatic foci at animal lung tissue sections. IHC protocol briefly started with the de-paraffinization in xylene and graded ethanol, sections were boiled in citrate buffer for 20 min, then permeabilized in TBST solution for 15 min at 25 °C. Then, sections were stained with the primary antibodies of Ki67 (Cat #9129, 1:200, Cell Signaling, Boston, MA, USA) or CD-31 (Cat #3528, 1:200, Cell Signaling) and diluted in blocking solution for 24 h at 4 °C. Next day, sections were rinsed and stained with secondary antibodies for 1 h. At the end of the experiment, images were captured at the Research Core Facility, LSUHSC, Shreveport, LA, USA, in 10× magnification power using an Olympus iXplore CSU W1 spinning disk confocal microscope (Center Valley, PA, USA). Experiments were performed in triplicate to ensure reproducibility and statistical significance.

### 4.11. Hematoxylin and Eosin Y (H&E) Staining

Tumor samples were fixed in 10% neutral buffered formalin for 48 h, transferred to 70% ethanol, and then paraffin-embedded. Paraffin-embedded tumor blocks were sectioned into 5 µm sections utilizing a Leica RM2035 microtome by AML Laboratories (Augustine, FL, USA). Sections were fixed on positively charged slides, xylene dewaxed, rinsed with alcohol, rehydrated in water; finally, slides were stained with H&E [29].

### 4.12. Analysis of Clinical SMYD2, STAT3 and EZH2 Genes Expression Data

The TCGA gene expression data were analyzed using UALCAN, which is a publicly available web tool able to perform in-depth analysis [33,34,35,36,37]. The mRNA expression patterns of SMYD2, STAT3, and EZH2 were analyzed. The gene expression profiling interactive analysis (GEPIA) was used to explore RNA sequence expression difference between normal and cancer samples [37]. GEPIA was used for differential expression analysis comparison of SMYD2, STAT3, and EZH2 in various cancers versus normal organ tissues [37].

### 4.13. Western Blot Analysis

Collected ZR-75-1 tumor samples were weighed, and total protein contents were extracted in RIPA lysis buffer (Thermo Fisher Scientific, Madison, WI, USA), supplemented with mammalian protease arrest (G-Biosciences, St. Louis, MO, USA), and were homogenized using an ultrasonic homogenizer (Qsonica Sonicator, Newtown, CT, USA). Homogenates were incubated at 4 °C for 4 h, then centrifuged for 15 min at 14,000× *g*, and supernatants were stored at −80 °C. The protein concentration was assessed by the Pierce BCA Protein Assay (Bio-Rad, Hercules, CA, USA). Lysates were loaded as 15 µg tumor lysate. Proteins were electrophoresed on Mini-PROTEAN TGX precast polyacrylamide gels (BIO-RAD) using Tris/glycine/SDS running buffer and transferred to Immuno-Blot PVDF membranes (BIO-RAD). Blotted membranes were blocked with 5% BSA (Cell Signaling Technology, Beverly, MA, USA) in TBST (10 mM Tris-HCl, 150 mM NaCl, 0.1% Tween-20) for 2 h with gentle rocking at rt. Immunoblots were incubated overnight at 4 °C with appropriate primary antibodies (Cell Signaling Technology). After incubation, the membranes were washed 5 times with TBST and then probed with HRP-labeled secondary antibodies (Cell Signaling Technology) for 1 h with agitation at rt, followed by rinsing 5 times with TBST. Proteins were detected using the ChemiDoc XRS chemiluminescent gel imaging system and analyzed using Image Lab software (Version 5.2.1, Bio-RAD, Hercules, CA, USA). All experiments were performed in triplicate, and β-tubulin was used as a housekeeping protein to confirm equal sample loading in all lanes.

### 4.14. Statistical Analysis

Data analysis was performed using GraphPad Prism software, version 8.0.2. (La Jolla, CA, USA). Results were presented as mean ± standard error of the mean (SEM) for continuous variables. Differences among various treatments and control groups in the animal study were determined using ordinary One-way ANOVA, followed by the post hoc Dunnett’s multiple comparisons test. A difference of * *p* < 0.05 was considered statistically significant, where: (* *p* < 0.05, ** *p* < 0.01, *** *p* < 0.001, and **** *p* < 0.0001). Combination data were analyzed, and results showed combination index (CI) values according to the median-effect principle, where CI < 1, =1, and >1 indicate synergism, additive effect, and antagonism, respectively [28,38]. CI values calculated as follows: CI = [Xc/X + Tc/T], where X and T stand for the concentrations of individual combination ingredients, OC and LA, that induced 50% cell growth inhibition (IC_50_); Xc and Tc are the concentrations of combination ingredients that induce 50% cell growth inhibition when used combined as determined by non-linear regression curve fit analysis [28,38].

## 5. Conclusions

This study reports the potential of EVOO phenolic secoiridoids for the control of BC progression and metastasis in female nude mice xenograft and tail vein models. Screening of EVOO phenolics library identified OC and LA as the most active anti-BC hits. OC-LA individual and combination treatments showed effective suppression of the luminal A BC ZR-75-1 cells tumor progression and the TNBC MDA-MB-231 cells metastasis and clonogenicity. The OC-LA combination significantly suppressed the expression of the lysine methyltransferases SMYD2 and EZH2 and reduced the activation of their downstream receptor tyrosine kinase STAT3, inhibiting its downstream mitogenic signaling pathway. Histopathological analysis of collected tumors and lung tissues showed significantly reduced metastatic foci, increased fibrosis, and mitotic figures in OC-LA combination-treated samples. This study findings validate the anti-BC activity of OC and LA, and their natural co-occurrence in EVOO would significantly enhance this activity. OC-LA combination could serve as a prospective future nutraceutical candidate to control invasive BC progression and metastasis.

## Figures and Tables

**Table 1:** Assessment of EVOO phenolics’ effects on the viability of the TNBC cells MDA-MB-231 and the luminal A BC cells ZR-75-1 at a single 10 µM treatment.

Compounds (10 µM)	MDA-MB-231 (48 h)			ZR-75-1 (48 h)		
	% Cell Viability	SEM	*p*-Value	% Cell Viability	SEM	*p*-Value
Tyrosol	99.24	2.76	ns	75.67	4.40	0.031
Hydroxytyrosol	95.94	3.21	ns	75.87	5.77	0.034
Pinoresinol	82.48	3.79	0.008	90.40	6.38	ns
Acetoxypinoresinol	78.29	2.36	0.001	87.26	7.60	ns
Oleocanthal	80.30	4.97	0.002	95.84	3.85	ns
Hydroxyoleocanthal	55.85	1.81	<0.001	85.17	2.93	ns
Ligstroside aglycone	63.43	3.29	<0.001	38.74	2.69	<0.001
Oleuropein	97.85	2.32	ns	103.36	5.82	ns
Oleuropein aglycone	84.52	3.73	0.030	82.24	5.07	ns

**Table 2:** Comparison of the in vitro antiproliferative activity of LA and OC versus standard anticancer drugs using MTT assay.

Compounds	BC Cell Lines, IC_50_	
	ZR-75-1	MDA-MB-231
PTX	4.1 nM	6.3 nM
GFT	5.6 µM	18.2 µM
TAM	5.8 µM	N/A
FUL	2.9 µM	N/A
OC	15.3 µM	23.0 µM
LA	3.9 µM	13.1 µM
BAY-598	18.6 µM	27.7 µM

## Data Availability

The data used to support the findings of this study can be made available by the corresponding author upon request.

## Supplementary Materials

The following supporting information can be downloaded at: https://www.mdpi.com/article/10.3390/molecules30153157/s1, Supplementary Figure S1: Comparison of the expression of SMYD2, STAT3, and EZH2 with normal tissues and different cancer types. Supplementary Figure S2: Clinical relevance of SMYD2, STAT3, and EZH2 in various cancer types, including BC. Supplementary Figure S3: The raw Western blotting images for ZR-75-1 primary tumors lysates obtained from female nude mouse xenograft model showing SMYD2, EZH2, STAT3, and p-STAT3 expression levels for LA and OC monotherapies, LA-OC combination, and VC.
