# Supplementary material for: Harnessing Nature’s Chemistry: Deciphering Olive Oil Phenolics for the Control of Invasive Breast Carcinoma
Source: Molecules. 2025 Jul 28;30(15):3157. doi: 10.3390/molecules30153157 (PMC12348913; doi:10.3390/molecules30153157)
Supplement: Supplementary file 1 [file molecules-30-03157-s001.zip › molecules-3727055-supplementary.pdf]

molecules

Article Supplementary Files

Harnessing Nature's Chemistry: Deciphering Olive Oil Phenolics for The Control of Invasive Breast Carcinoma

Nehal A. Ahmed [1,2], Abu Bakar Siddique [1,2], Afsana Tajmim, [1] Judy Ann King [3], and Khalid A. El Sayed [1,*]

1 Department of Basic Pharmaceutical and Toxicological Sciences, College of Pharmacy, University of Louisiana at Monroe, 1800 Bienville Drive, Monroe, LA 71201, USA; atefkhaledahmedabdn@warhawks.ulm.edu (N.A.A); siddique.ulm@gmail.com (A.B.S.); afsana.ulm@gmail.com (A.T.)
2 Both authors contributed equally
3 Foundational and Clinical Sciences Department, Thomas F. Frist, Jr. College of Medicine, Belmont University, 1900 Belmont Boulevard, Nashville, TN 37212, USA; judy.king@belmont.edu (J.A.K.)

* Correspondence: elsayed@ulm.edu (K.A.E.); Tel.: +1-318-342-1725

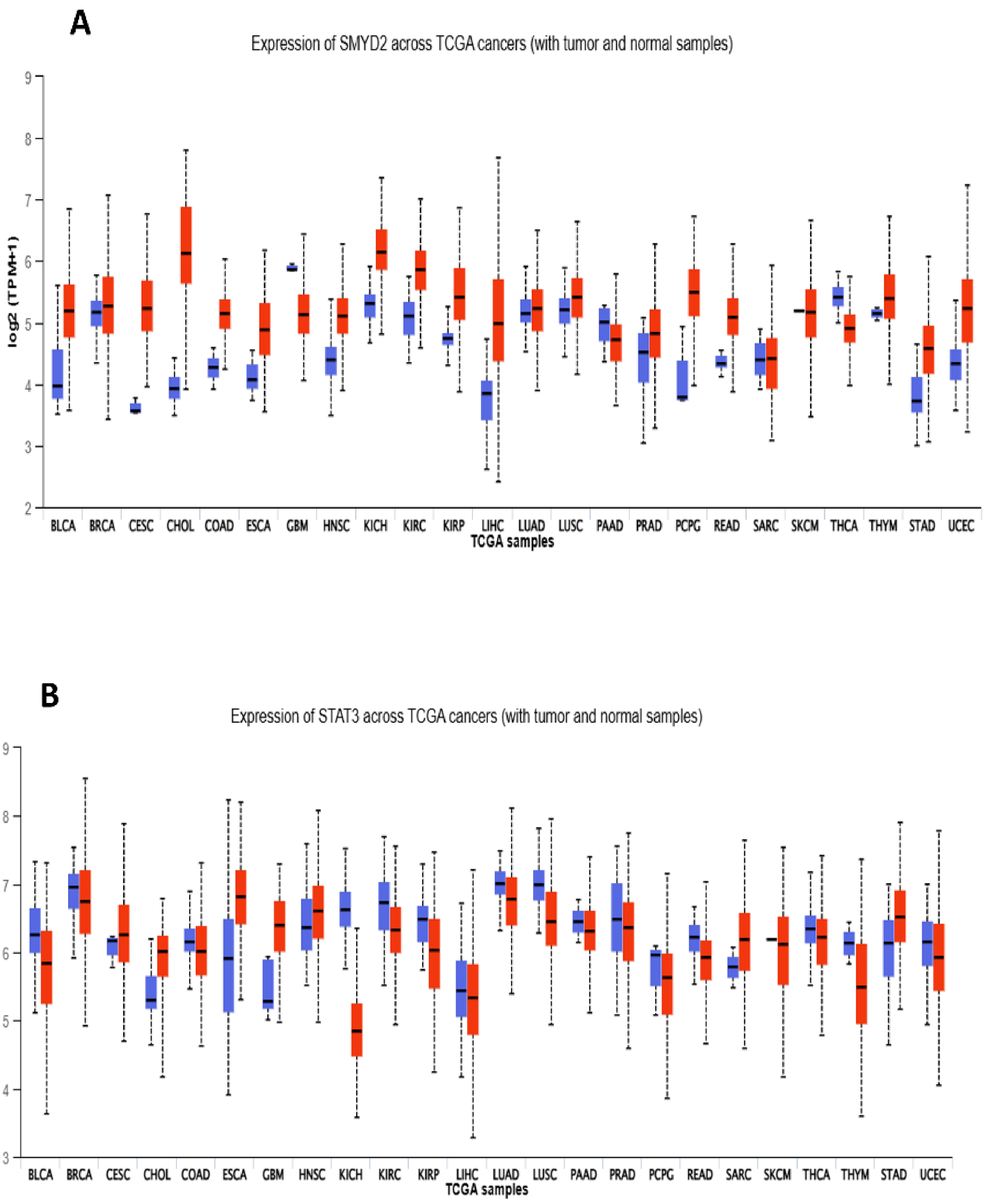
A
Expression of SMYD2 across TCGA cancers (with tumor and normal samples)
log2 (TPM+1)
9
8
7
6
5
4
3
2
BLCA BRCA CESC CHOL COAD ESCA GBM HNSC KICH KIRC KIRP LIHC LUAD LUSC PAAD PRAD PCPG READ SARC SKCM THCA THYM STAD UCEC
TCGA samples
B
Expression of STAT3 across TCGA cancers (with tumor and normal samples)
log2 (TPM+1)
9
8
7
6
5
4
3
BLCA BRCA CESC CHOL COAD ESCA GBM HNSC KICH KIRC KIRP LIHC LUAD LUSC PAAD PRAD PCPG READ SARC SKCM THCA THYM STAD UCEC
TCGA samples

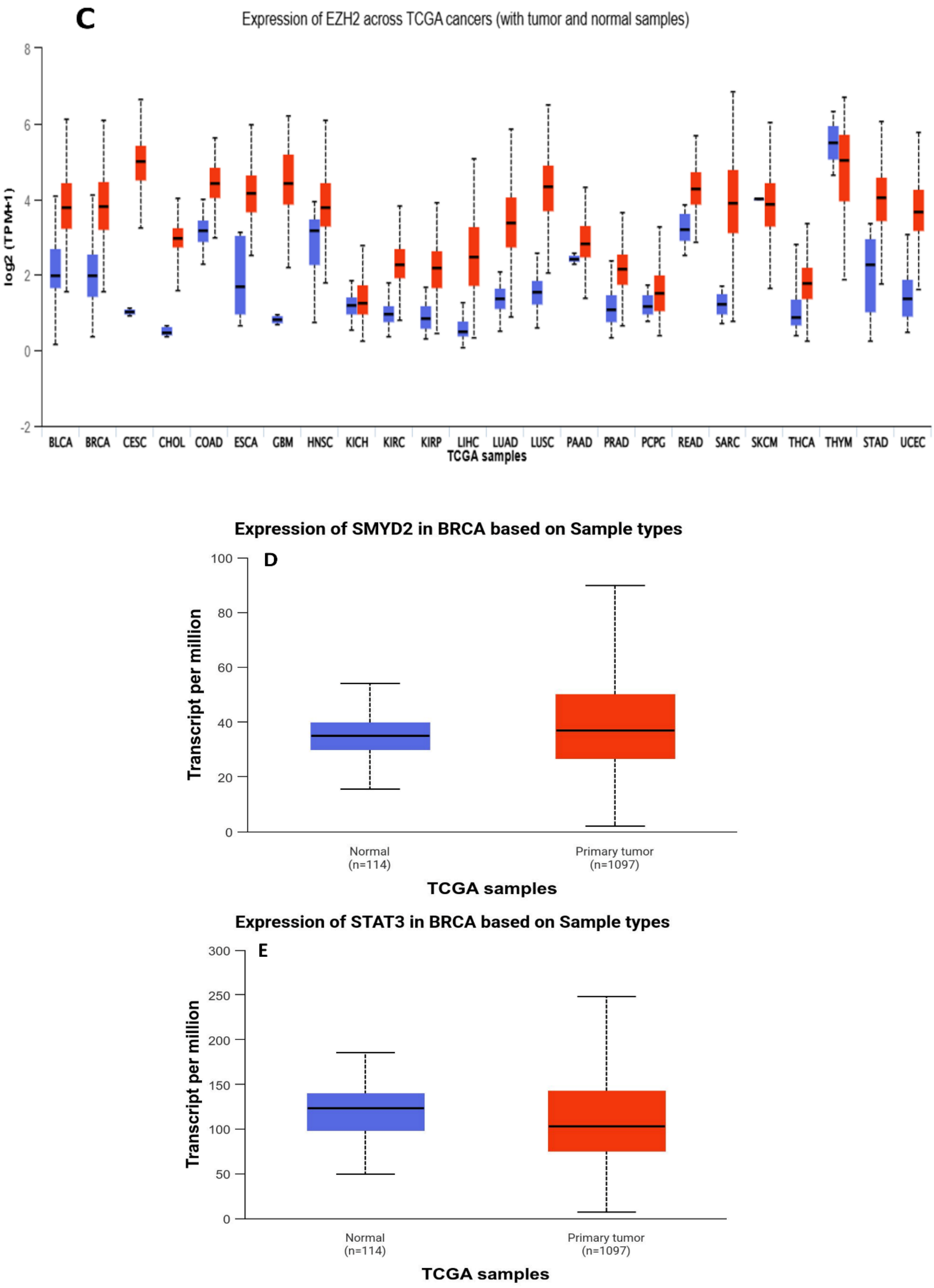
C
Expression of EZH2 across TCGA cancers (with tumor and normal samples)
log2 (TPM+1)
8
6
4
2
0
-2
BLCA BRCA CESC CHOL COAD ESCA GBM HNSC KICH KIRC KIRP LIHC LUAD LUSC PAAD PRAD PCPG READ SARC SKCM THCA THYM STAD UCEC
TCGA samples
Expression of SMYD2 in BRCA based on Sample types
D
Transcript per million
100
80
60
40
20
0
Normal
(n=114)
Primary tumor
(n=1097)
TCGA samples
Expression of STAT3 in BRCA based on Sample types
E
Transcript per million
300
250
200
150
100
50
0
Normal
(n=114)
Primary tumor
(n=1097)
TCGA samples

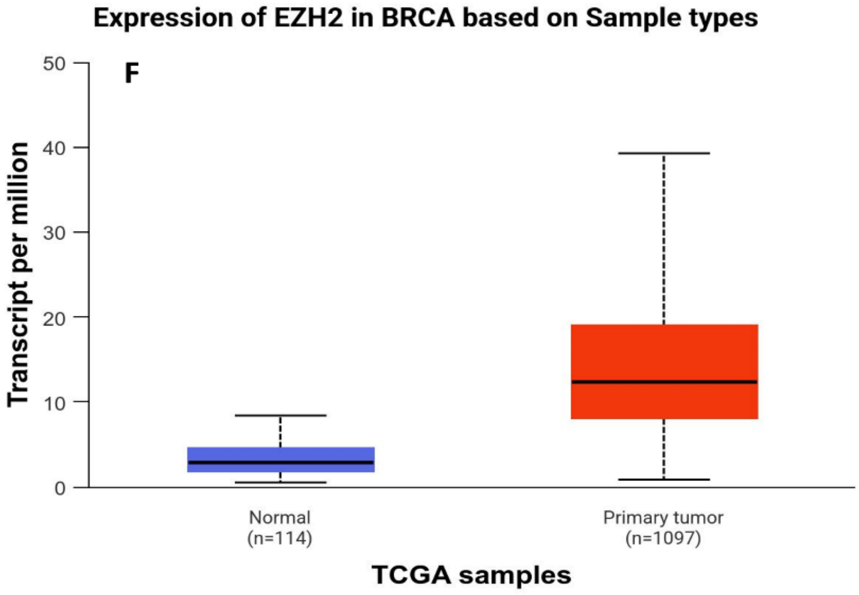

Supplementary Figure S1. Comparison of the expression of SMYD2, STAT3, and EZH2 with normal tissues and different cancer types. (**A**-**C**) mRNA expression levels of SMYD2, STAT3, and EZH2, respectively, across various cancer types in The Cancer Genome Atlas (TCGA) dataset. The box plot compares mRNA expression ($\log_2$[TPM+1]) between tumor (red) and corresponding normal (blue) tissue samples across 24 TCGA cancer types. (**D**-**F**) mRNA expression levels of SMYD2, STAT3 and EZH2, respectively, in invasive breast carcinoma (BRCA) based on sample type from TCGA. The box plot compares transcript levels (transcripts per million, TPM) between normal breast tissue samples (blue, *n*=114) and primary breast tumor samples (red, *n*=1097) [33-37].

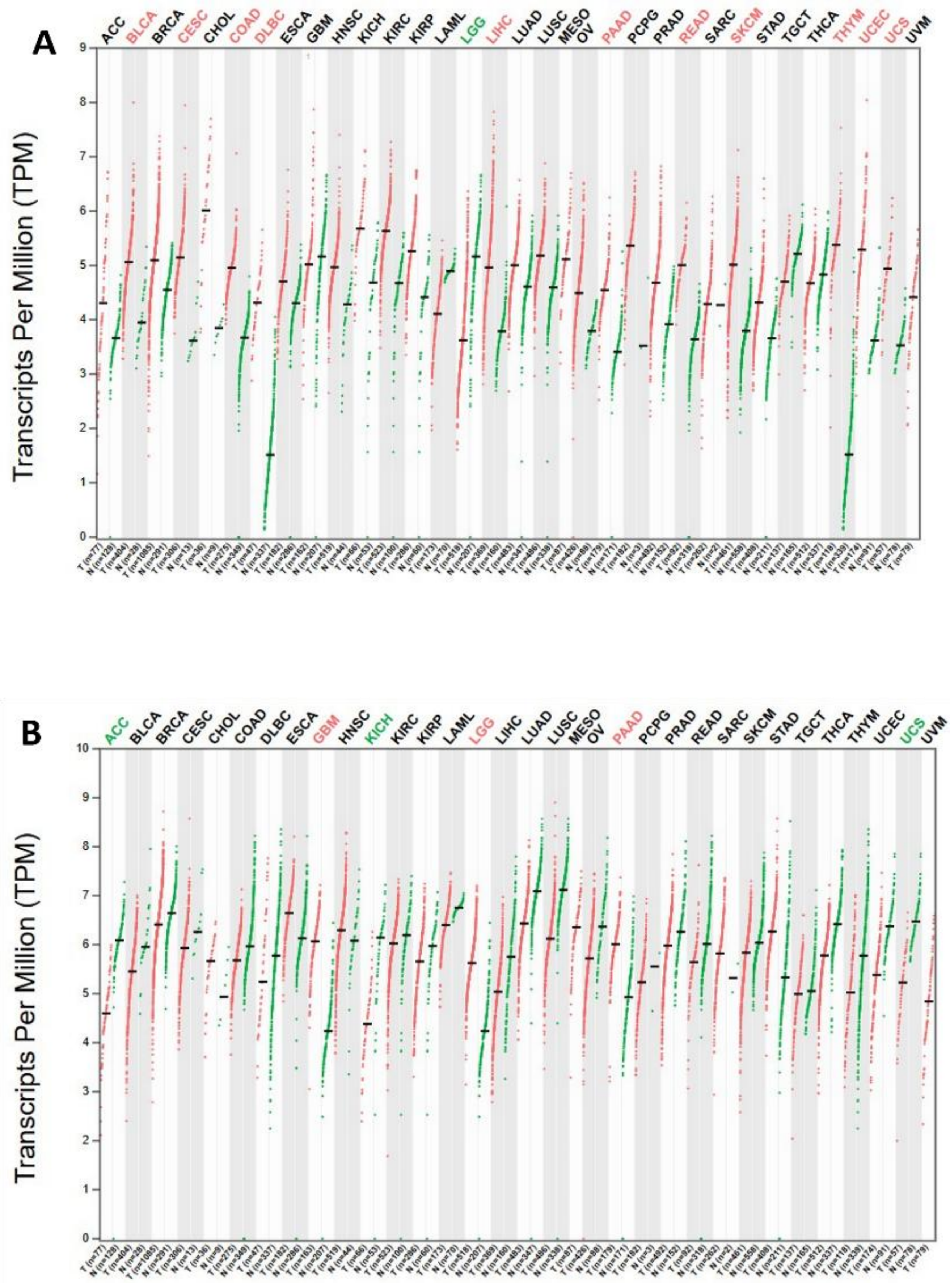
A
Transcripts Per Million (TPM)
ACC BLCA BRCA CESC CHOL COAD DLBC ESCA GBM HNSC KICH KIRC KIRP LAML LGG LIHC LUAD LUSC MESO OV PAAD PCPG PRAD READ SARC SKCM STAD TGCT THCA THYM UCEC UCS UVM
B
Transcripts Per Million (TPM)
ACC BLCA BRCA CESC CHOL COAD DLBC ESCA GBM HNSC KICH KIRC KIRP LAML LGG LIHC LUAD LUSC MESO OV PAAD PCPG PRAD READ SARC SKCM STAD TGCT THCA THYM UCEC UCS UVM

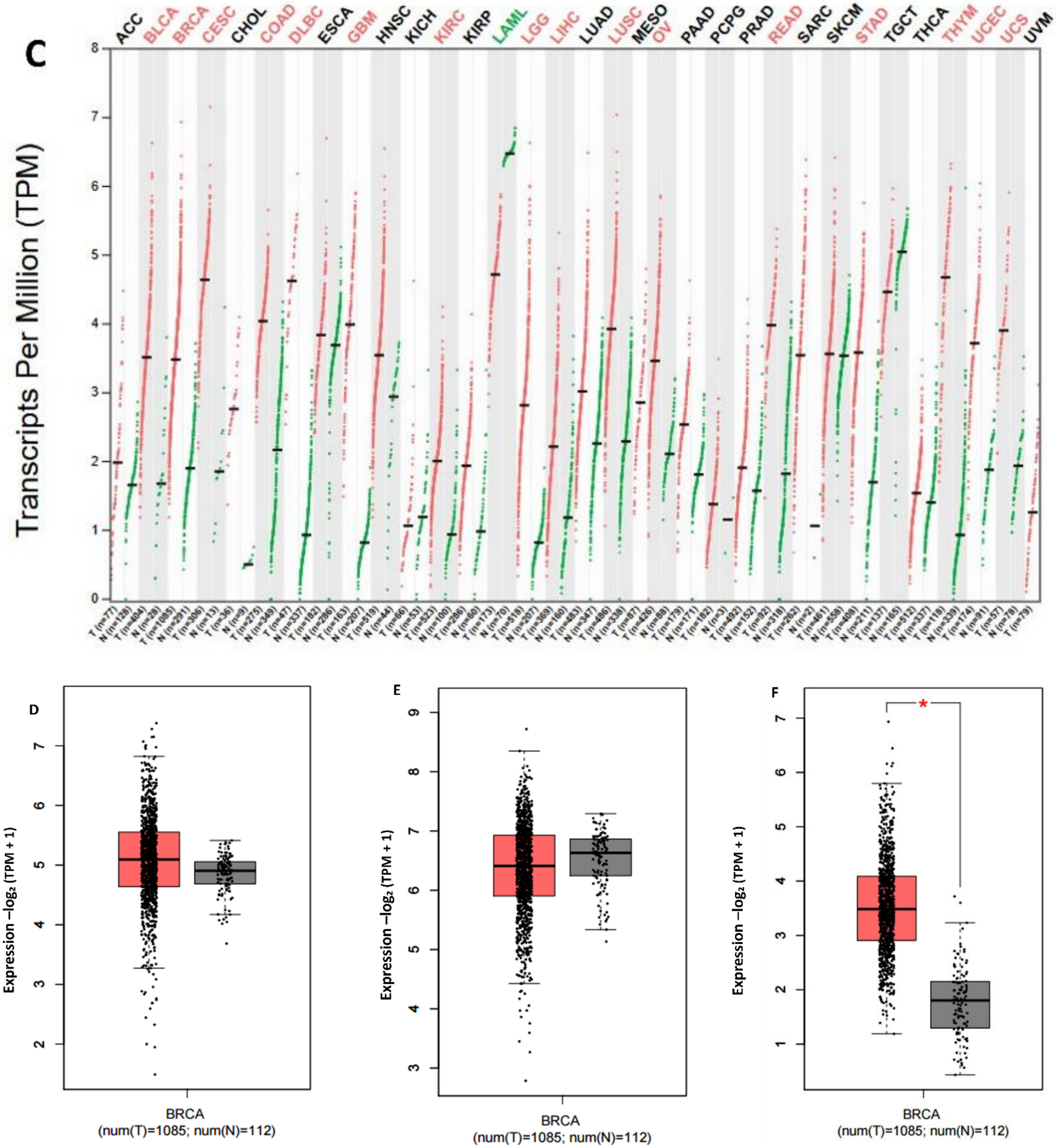

Supplementary Figure S2. Clinical relevance of SMYD2, STAT3, and EZH2 in various cancer types including BC. (**A**-**C**) mRNA expression levels of SMYD2, STAT3, and EZH2, respectively, across various cancer types in The Cancer Genome Atlas (TCGA) dataset. The box plot compares mRNA expression (log_2[TPM+1]) between BC (red) and corresponding normal (blue) tissue samples across 24 TCGA cancer types. (**D**-**F**) The mRNA expression levels of SMYD2, STAT3 and EZH2, respectively, in invasive breast carcinoma (BRCA) based on sample type from TCGA. The box plot compares transcript levels (transcripts per million, TPM) between normal breast tissue samples (blue, n=114) and primary breast tumor samples (red, n=1097) [33-37].

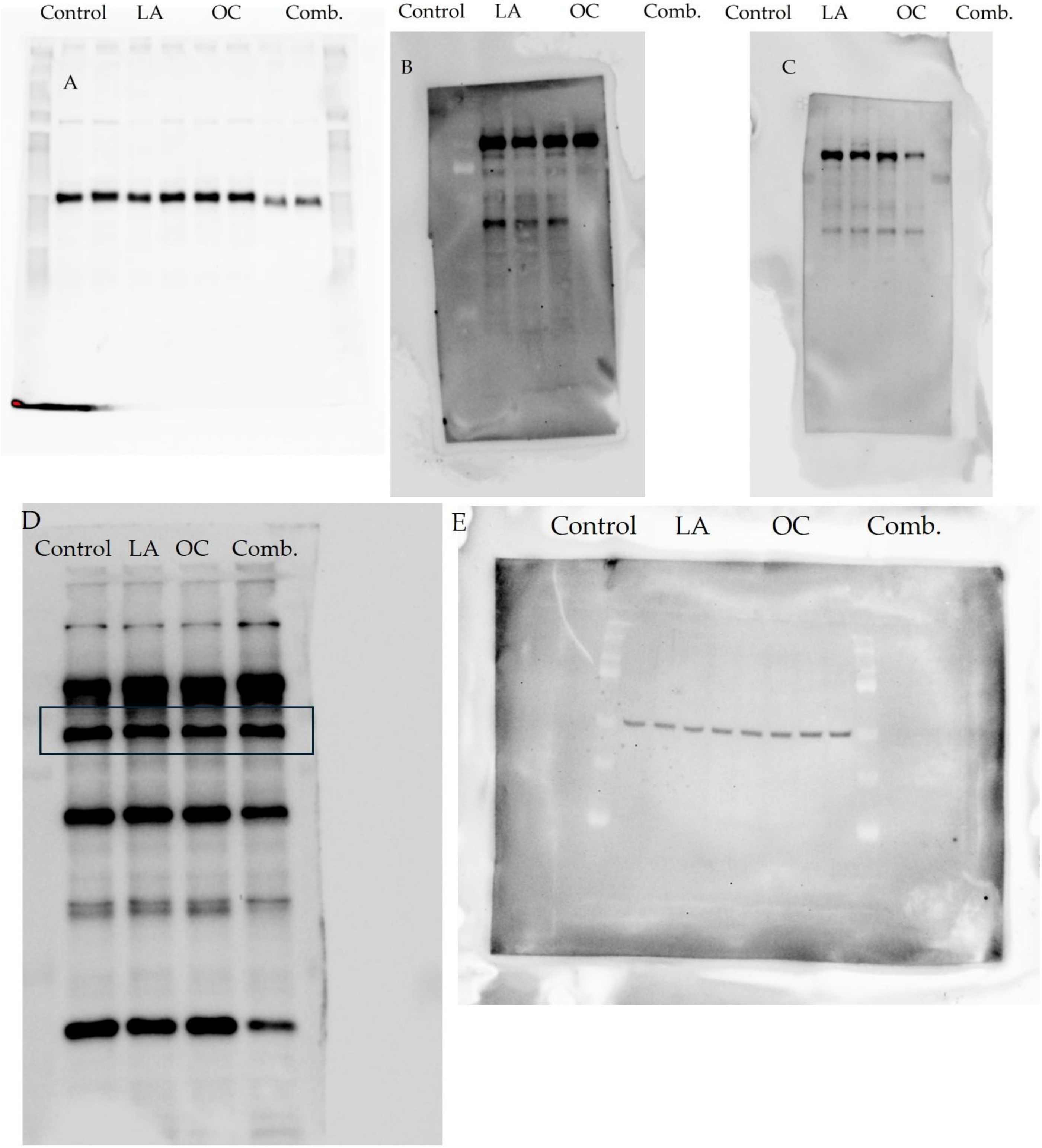

Supplementary Figure S3. The raw Western blotting images for ZR-75-1 primary tumors lysates obtained from female nude mouse xenograft model showing SMYD2, EZH2, and p-STAT3 expression levels for LA and OC monotherapies, LA-OC combination, and vehicle control. (**A**) SMYD2 protein expression level normalized to β-tubulin. (**B**) p-STAT3 protein expression level normalized to β-tubulin. (**C**) EZH2 protein expression level normalized to β-tubulin. (**D**) Total STAT3 expression level. (**E**) β-tubulin protein expression level as a loading control.
